# Thioredoxin-1 regulates self-renewal and differentiation of murine hematopoietic stem cells through p53 tumor suppressor

**DOI:** 10.1186/s40164-022-00329-3

**Published:** 2022-10-31

**Authors:** Shaima Jabbar, Parker Mathews, Xiaobei Wang, Pasupathi Sundaramoorthy, Emily Chu, Sadhna O. Piryani, Shengli Ding, Xiling Shen, Phuong L. Doan, Yubin Kang

**Affiliations:** 1 Division of Hematologic Malignancies and Cellular Therapy, Department of Medicine, School of Medicine, Duke University Medical Center, 2400 Pratt Street, Suite 5000, Durham, NC DUMC 396127710 USA; 2 Department of Biomedical Engineering, Pratt School of Engineering, Duke University, Durham, NC 27710 USA; 3 Duke Cancer Institute, Duke University, Durham, NC USA

**Keywords:** Hematopoietic stem cells, Radiation mitigation, Hematopoietic stem cell transplant, Reactive oxygen species, p53, Redox homeostasis

## Abstract

**Background:**

Thioredoxin-1 (TXN1) is one of the major cellular antioxidants in mammals and is involved in a wide range of physiological cellular responses. However, little is known about the roles and the underlying molecular mechanisms of TXN1 in the regulation of hematopoietic stem/progenitor cells (HSPCs).

**Methods:**

TXN1 conditional knockout mice (ROSA-CreER-TXN1^fl/fl^) and TXN1^fl/fl^ control mice were used. The mice were treated with tamoxifen and the number and biological functions of HSPCs were measured by flow cytometry, PCR and western blot. Limiting dilution competitive transplantation with sorted HSCs and serial transplantations were performed to assess the effects of TXN1 knockout on HSC self-renewal and long-term reconstitutional capacity. RNA sequencing (RNA-seq) was performed to investigate the downstream molecular pathways of TXN1 deletion in murine HSPCs. CRISPR/Cas9 knockout experiments were performed in vitro in EML murine hematopoietic stem/progenitor cell line to investigate the effects of TXN1 and/or TP53 deletion on cell survival, senescence and colony forming units. TP53 protein degradation assay, CHiP PCR and PGL3 firefly/renilla reporter assay were performed. The effects of TXN1 on various molecular pathways relevant to HSC radiation protection were examined in vitro and in vivo.

**Results:**

TXN1-*TP53* tumor suppressor axis regulates HSPC biological fitness. Deletion of TXN1 in HSPCs using in vivo and in vitro models activates *TP53* signaling pathway, and attenuates HSPC capacity to reconstitute hematopoiesis. Furthermore, we found that knocking out of TXN1 renders HSPCs more sensitive to radiation and treatment with recombinant TXN1 promotes the proliferation and expansion of HSPCs.

**Conclusions:**

Our findings suggest that TXN1-*TP53* axis acts as a regulatory mechanism in HSPC biological functions. Additionally, our study demonstrates the clinical potential of TXN1 for enhancing hematopoietic recovery in hematopoietic stem cell transplant and protecting HSPCs from radiation injury.

**Supplementary Information:**

The online version contains supplementary material available at 10.1186/s40164-022-00329-3.

## Introduction

Hematopoietic stem cells (HSCs) are a rare population of cells that are responsible for continuous production and maintenance of all lineages of blood and immune cells throughout the lifespan [1, 2]. HSCs are characterized by their capacities of self-renewal, quiescence, proliferation and differentiation. These functions of HSCs are orchestrated by extrinsic signals in bone marrow (BM) microenvironment niches such as oxygen tension as well as by intrinsic regulatory mechanisms including DNA damage responses, epigenetic modification, transcription factors, metabolic alteration, mitochondrial fitness and morphogenic signaling molecules [2, 3]. Despite significant advances in our understanding of HSC biology over the last several decades, the detailed molecular mechanism and pathways regulating HSC functions remain incompletely characterized.

Thioredoxin system is one of the major cellular antioxidant systems in the aerobic organisms and consists of thioredoxin (TXN1), thioredoxin reductase, and nicotinamide adenine dinucleotide phosphate (NADPH) that serves as an electron donor to recycle oxidized TXN1 to its reducing form) [4–6]. TXN1 provides a reducing equivalent that supports a variety of cell biological functions including cell survival, cell proliferation, and maintenance of redox homeostasis. Compared to other known reducing systems in the cell, TXN1 is the only protein that maintains the reducing power for the ribonucleotide reductase enzyme, which is the building block for DNA replication and repair [4, 6]. Besides functioning as a direct and indirect antioxidant [6–9], TXN1 is involved in a wide range of physiological cellular responses independent of reactive oxygen species (ROS) [10–13]*.* For instance, human TXN1 was originally found as a soluble growth factor for human T cell leukemia virus type I-transformed cells [14, 15] or EBV-transformed B cells [16]. TXN1 is produced by all cell types including hepatocytes, fibroblasts, activated monocytes, and lymphocytes [17, 18], and can function in a chemokine-like manner to induce cell migration [19, 20] and cell proliferation [16, 20–23]. Recent studies have also provided evidence that thioredoxin system balances bioenergetics and biosynthetic demands and regulates the proliferation and activation of immune cells like T cells, natural killer cells (NKs) and macrophages [24–27].

Using a mass-spectrometry based semi-quantitative proteomics screen, we previously reported that TXN1 was significantly upregulated in the bone marrow of hematopoietic stem cell transplant (HSCT) recipient mice treated with AMD3100 (Mozobil, plerixafor) relative to transplant recipient mice given control PBS buffer [28]. AMD3100, a CXCR4 antagonist, improved hematopoietic recovery following myeloablative HSCT in our mouse model and in patients receiving myeloablative allogeneic transplants [28, 29]. We have also shown that recombinant TXN1 has marked protective and proliferative effects on HSCs in mouse models of HSCT and radiation injury [30]. Ex-vivo culture of murine HSCs with recombinant TXN1 enhances HSC long-term repopulation capacity and administration of recombinant TXN1 up to 24 h following lethal total body irradiation (TBI) rescues BALB/c and C57Bl/6 mice from radiation-induced lethality [30, 31]. However, the molecular mechanisms underlying the protective effects of TXN1 on HSCs remain unclear.

*TP53* tumor suppressor gene is one of the most important genes that guards the stability and integrity of HSC genome [32, 33]. Upon exposure to genotoxic stress, *TP53* arrests the cell cycle and can induce cellular apoptosis to protect HSCs from mutational accumulation [34, 35]. On the other hand, TP53-activated apoptosis pathway could attenuate HSCs’ self-renewal and differentiation capacity, resulting in hematopoietic failure. Therefore, inhibiting *TP53* may protect HSCs against radiation-induced injury. Indeed several lines of studies have found that *TP53* deficiency protects hematopoietic system after radiation exposure, and ameliorates radiation induced bone marrow (BM) injury, although constitutive inhibition of *TP53* in HSCs causes premature exhaustion of HSCs and increases the incidences of hematologic malignancies [32, 33, 36]. Molecules and pathways that regulate HSC functions through *TP53* pathway provide an attractive alternative approach to directly targeting TP53 for enhancing stem cell function in HSCT and in radiation injury.

In this current study, we determined the molecular mechanisms through which TXN1 regulates HSC functions in normal conditions and under conditions of stress such as radiation injury. We developed a TXN1 conditional knockout mouse model because constitutive homozygous deletion of TXN1 is embryonically lethal [37]. Additionally, EML murine hematopoietic stem/progenitor cell line was used for in vitro genetic studies. We found that deletion of TXN1 impaired the reconstitution and differentiation of HSCs through up-regulation of the *TP53* apoptosis signaling pathway. Our study provides a new insight into the roles of TXN1 in HSC functions and the molecular mechanisms through which TXN1 regulates HSCs. Furthermore, our findings demonstrated the clinical application potential of using TXN1 for radiation protection and for enhancing hematopoietic recovery in HSCT.

## Materials and methods

### Animal experiments

Mice used in this study were purchased from the Jackson Laboratory (Bar Harbor, ME) or bred in-house. The mice were housed in our specific pathogen-free facility and maintained at 23–25 °C on a 12 h day / 12 h dark cycle and were provided with autoclave food and water ad libitum. For total body irradiation, animals were irradiated with a Mark I ^137^Cs Irradiator (JL Shephard, San Fernando, CA). All animal studies were conducted in accordance with guidelines approved by the Institutional Animal Care and Use Committee (IACUC) at Duke University (Protocol A097-17–04).

### Generation of ROSA-CreER-TXN1^fl/fl^ mice

Breeding pair of TXN1^fl/fl^ mice were obtained from the Jackson Laboratory (stock 30,221) [79]. These TXN1^fl/fl^ mice have *loxP* sites flanking Txn1 exons 2 and 3. When bred with mice that express a Cre recombinase, the floxed region is deleted in Cre expressing tissues, creating a protein null allele. VAV-Cre (8610), and ROSA-CreER (8463) mice were purchased from the Jackson Laboratory (Bar Harbor, ME). TXN^fl/fl^ mice were bred with ROSA-CreER to generate ROSA-CreER-TXN1^fl/fl^ mice. Unless otherwise noted, all murine models in this study were bred on a C57BL/6 genetic background. Inbred sex-matched and age-matched TXN1^fl/fl^ and ROSA-CreER*-*TXN1^fl/fl^ mice (6–8 weeks old) mice were used for experiments.

### Genotyping

Genomic DNA was isolated from tail clips in DirectPCR lysis buffer (Viagen, Los Angeles, CA). All genotyping reactions were run with GoTaq Flexi DNA Polymerase (Promega, Madison, WI). Primer sequences follow: ROSA WT-F: CTGGCTTCTGAGGACCG; ROSA WT-R: CCGAAAATCTGTGGGAAGTC; ROSA MT-F: CGTGATCTGCAACTCCAGTC; ROSA MT-R: AGGCAAATTTTGGTGTACGG; TXN1-F: GCACCCAAATGGGAGAGTC; TXN1-R: ACCAAGAAGCGTTAGAACTGG.

### Tamoxifen treatment

To induce *TXN1* deletion in vivo, tamoxifen (Sigma Aldrich, St. Louis, MO) was dissolved in corn oil with stirring overnight at 37˚C and was then administered by an intra-peritoneal injection into ROSA CreER-TXN1^fl/fl^ mice or TXN^fl/fl^ mice at a dose of 75 mg/kg every day for five consecutive days. The mice were euthanized and the phenotype was investigated at day 10 after injection of tamoxifen, unless otherwise indicated.

### Flow cytometry

Bone marrow cells were flushed from axial bones (femurs and tibias) of mice and were suspended in FACS buffer (2% FBS in PBS). Whole BM was lysed in ACK Lysis Buffer (Lonza) and washed with FACS buffer. 1 × 10^6^ cells were used and stained with fluorochrome-conjugated surface marker antibodies from BD Bioscience, BioLegend and Miltenyi Biotech, unless otherwise indicated. To characterize hematopoietic stem cells and progenitors, bone marrow cells were stained with anti-Scal-1 (D7), anti-cKit (2B8), anti-CD150 (TC15-12F12.2) and CD48 (HM48.1). To assess progenitor sub-populations, BM cells were stained with Anti-CD34 (HM34) and CD16/32 (93) for CMPs, MEPs and GMPs, and anti-CD127 (A019D5) and Flt3 (A2F10) for CLPs, respectively, and subjected to live cell flow cytometry. All flow cytometry was performed on a BD FACSCanto II flow cytometer (BD Bioscience) and the data was analyzed with FlowJo_v10.8.1.

### Colony-forming cell (CFC) assay

CFC assays were performed by culturing total bone marrow cells (2 × 10^4^ / dish) collected from ROSA CreER-TXN1^fl/fl^ and TXN1^fl/fl^ control mice in triplicate in 35-mm petri dishes (Falcon) containing 2 mL MethoCult GF M3434 medium (Stem Cell Technologies). After 12 days of incubation at 37 °C in 5% CO_2_, colonies were manually counted under a microscope.

### Reactive oxygen species (ROS) and JC-1 (mitochondrial membrane potential) assays

The MACS magnetic cell separation system (Miltenyi Biotech) was used to enrich mouse Lineage negative (Lin-) cells from whole bone marrow. The purity of mouse Lin- BM cells were between 90 and 98% and confirmed by flow cytometry prior to downstream analysis. Lin- cell were used for ROS and JC-1 assays. 5 × 10^5^ Lin- cells were incubated with 10 µM 6-carboxy-2',7'-dichlorodihydrofluorescein diacetate 9 (carboxy-H2DCFDA, Invitrogen) and 2 μM JC-1 (Invitrogen) at 37 °C, 5% CO_2_, for 20 min. Immediately following incubation, a minimum of 10,000 events were acquired with a BD FacsCanto II flow cytometer and the data was analyzed with the FlowJo software package.

### Annexin V apoptosis assay

Isolated Lin- cell from ROSA CreERTXN1^fl/fl^ mice or TXN^fl/fl^ mice were stained with Annexin V (BD Bioscience) according to manufacturer’s instructions and analyzed by flow cytometry. Briefly, 5 × 10^4^ Lin- cell were suspended with 1X binding buffer and stained with 5µL of Annexin V and 10 µL of propidium iodide and incubated for 15 min at RT in dark. After washing, the cells were analyzed and a minimum of 10,000 events were acquired for analysis.

### Senescence β-galactosidase assay

5 × 10^5^ Lin- cells from ROSA CreER-TXN1^fl/fl^ mice or TXN^fl/fl^ mice were stained with the CellEvent™ Senescence Green Flow Cytometry Assay Kit according to manufacturer’s instructions (Invitrogen). A minimum of 10,000 events were acquired with a BD FacsCanto II flow cytometer and the data was analyzed with the FlowJo software package.

### Seahorse measurement of oxygen consumption and cellular ATP concentration

Total bone marrow cells were isolated from ROSA CreER-TXN1^fl/fl^ mice or TXN^fl/fl^ mice as described earlier. 2.5 × 10^5^ cell were seeded per well in XF24 microplate and incubated under normal cell culture conditions prior to analysis. The cell metabolic rates and oxygen-consumption rate were measured following the sequential addition of 1 × 10^−6^ M oligomycin (Sigma 75,351), 4 × 10^−6^ M carbonyl cyanide 4-(trifluoromethoxy)- phenylhydrazone (FCCP; Sigma, C2920), 0.5 × 10^−6^ M rotenone (Sigma, R8875), and 0.5 × 10^−6^ M antimycin A (Sigma, A8674) on the Seahorse XF24 analyzer according to the manufacturer’s protocol.

### Western blotting

Cells were lysed on ice with Pierce™ IP Lysis Buffer (ThermoFischer), which contains 25 mM Tris•HCl pH 7.4, 150 mM NaCl, 1% NP-40, 1 mM EDTA, 5% glycerol, supplemented with protease inhibitor (Thermo Scientific) and phosphatase inhibitor (Thermo Scientific). Cell debris was then removed by spinning for 5 min at 4 °C. Protein concentrations were determined using the Pierce BCA Protein Assay Kit (Thermo Scientific). Whole cell extracts (50 μg of proteins) were fractionated by SDS-PAGE and transferred to a nitro cellular membrane using a transfer apparatus according to manufacturer’s instructions (Bio-Rad). Membranes were blocked with LICOR blocking buffer, washed and incubated with primary antibodies (1:1000 in blocking buffer) at 4 °C for 12 h. After washing, membranes were incubated with a 1:10,000 dilution (in blocking buffer) of fluorescent 700 or 800 anti-rabbit or anti-mouse antibodies for 1 h at room temperature. Blots were washed with TBST five times and scanned using a LICOR machine. All antibodies for immunoblotting used in this study can be found in the Key Resources Table 1.

**Table 1:** Summary of the reagents and materials used in this study

Reagent or resource	Source	Identifier
Antibodies		
Ms Cd45.1 PE Clone A20	BD Biosciences	Cat#553776
Ms Cd45.2 FITC Clone 104	BD Biosciences	Cat#553772
Ms Cd11b PE CloneM1/70	BD Biosciences	Cat#557397
Ms Ly-6G/Ly-6C (Sca1) PE Clone RB6-8C5	BD Biosciences	Cat#553128
Ms CD45R/B220 APC Clone RA3-6B2	BD Biosciences	Cat#553092
Ms CD3 APC-Cy7 Clone 17A2	BD Biosciences	Cat#560590
Ms CD117 (c-Kit) Clone 2B8	Biolegend	Cat#105811
Ms CD150 (SLAM) Clone TC15-12F12.2	Biolegend	Cat#115903
Ms CD48 Clone HM48-1	Biolegend	Cat#103403
Ms CD34 Clone HM34	Biolegend	Cat#128609
Ms CD16/32 Clone 93	Biolegend	Cat#101327
Ms CD127 (IL-7Ra) Clone A019D5	Biolegend	Cat#351311
Ms CD135 (Flt3) Clone A2F10	Biolegend	Cat#135309
Rabbit monoclonal to PUMA	Abcam	Cat#ab9643
Mouse monoclonal to NOXA	Abcam	Cat#ab13654
Rabbit polyclonal to p53	Abcam	Cat#ab131442
H2AX rabbit mAb	Cell signaling	Cat#D17A3
Monoanti Flag M2	Sigma Aldrich	Cat# F1804
TXN1 polyAb	ThermoFischer	PA587014
Beta actin Ab	Santa Cruz	Cat#sc4778
MDM2 mouse Ab	Santa Cruz	Cat#sc965
TXN2 Ab	Santa Cruz	Cat#sc133201
P21 Ab	Santa Cruz	Cat#sc6246
P16 Ab	Santa Cruz	Cat#sc166760
Chemicals, peptides, and recombinant proteins		
Tamoxifen	Sigma Aldrich	Cat#T5648; CAS 10540-29-1
Recombinant Human Thioredoxin-1	R&D Systems	Cat#1970-TX
PenStrep	Gibco	Cat#15140-122
Direct PCR	Viagen Biotech	102-T
Iscoves DMEM1X	Corning	10016cv
RPMI 1640 1X	Corning	10040cv
FBS Gemini	GeminiBio	100106
Lipofectamin RNA iMAX	Life technology	13778150
LE Agarose	Millipore Sigma	Cat#E-3120
Critical commercial assays		
MACS Ms lineage- selection kit	Miltenyi Biotech	Cat#130-090-858
Annexin V Apoptosis Assay	BD Biosciences	Cat#556547
ROS assay (H2DCFDA)	Invitrogen	Cat#D399
MitoProbe JC-1 assay	Invitrogen	Cat#M34152
CellEvent Senescence Green	Invitrogen	Cat#C10841
Experimental models: cell lines		
Mouse: EML, Clone 1	ATCC	ATCC: CRL-11691
Experimental models: organisms/strains		
Mouse: *Txn1*^*tm1.1Ees*^/J	Jackson Laboratory	JAX: 030221
Mouse: B6.Cg-*Commd10*^*Tg(Vav1-icre)A2Kio*^/J	Jackson Laboratory	JAX: 008610
Mouse: B6.129-*Gt(ROSA)26Sor*^*tm1(cre/ERT2)Tyj*^/J	Jackson Laboratory	JAX: 008463
Mouse: B6.SJL-*Ptprc*^*a*^* Pepc*^*b*^/BoyJ	Jackson Laboratory	JAX: 002014
Oligonucleotides		
Genoyping primers for ROSA-TXN mice (see Table 2)	IDT DNA	JAX genotyping protocols (see above)
Software and algorithms		
GraphPad Prism6	https://www.graphpad.com/	
FlowJo™ v10.8.1	https://www.flowjo.com/solutions/flowjo/downloads	
The R Project for Statistical Computing	https://www.r-project.org/	
Quant studio 6 flex	https://www.thermofisher.com/us/en/home/life-science/pcr/real-time-pcr/real-time-pcr-instruments/quantstudio-systems/models/quantstudio-6-7-pro.html	

### RNA-seq

RNA-Seq library preparation and sequencing was carried out at the Duke Genomic Center (Duke University, North Carolina). RNA integrity was rechecked by Duke core facility prior to sequencing. The Ribo-Zero Gold Kit was optimized for removal of all sizes of rRNA. Total RNA samples (2 μg) were mixed with the rRNA Removal Reagents in solution for 25 min. The mixture was then added to Ribo-Zero Gold Microspheres and incubated for 20 min followed by removal of the Microspheres with a spin-filter column (2 min). The rRNA-depleted RNA was recovered by a column-purification method. For this analysis, total RNA was isolated from Lin- HSCs from three tamoxifen-treated Rosa-CreER-TXN1^fl/fl^ mice and three tamoxifen-treated TXN^fl/fl^ mice by using Qiagen kit (cat# 74106), and 2 μg was used as input for the Ribo-Zero Gold Kit. Following rRNA removal, the RNA was used to prepare cDNA libraries using the ScriptSeq™ mRNA-Seq Library Preparation Kit (Epicentre). Libraries were sequenced on Illumina® GAIIx and HiSeq 2000 platforms.

### Bioinformatics

After sequencing, reads were mapped to the reference mouse genome (GRCm38/mm10) using TopHat v2.0. CuffDiff v2.2 to determine differential genes and transcript expression. To analyze differential expression, cummeRbund was used to look at overall changes in gene expression as well as differences in individual genes. Data were subjected to principal components analysis (PCA) to determine clustering patterns [80, 81]. We used R and Bioconductor to tie together the workflow and provide data structures. Genes with greater than 2 log2-fold change and FPKM values > 1 were selected for gene enrichment analyses using Database for Annotation. Ingenuity Pathway Analysis (IPA; www.qiagen.com/ingenuity; Qiagen; Redwood City, CA) was used to integrate genes and biological pathways.

We used DAVID platform and Ingenuity pathways analyses (IPA) to analyze the biological functions and categorize the gene sets (https://david.ncifcrf.gov/) [82]. In addition, we used Gene Set Enrichment Analysis (GSEA) software [83] that computes a Normalized Enrichment Score (NES) to give us an idea about the set of genes that are more upregulated or downregulated in our data set. We followed the reference [83] to set up *p* and q values to calculate the False Discovery Rate (FDR; q-value) [83]. For heat map designing, we used R software and we set up the FPKM and TPM values as a matrix to run heatmap2 and CS heat map codes.

### RT-PCR

One µg RNA was used to obtain cDNA by using High Capacity cDNA Reverse Transcription Kit (Life Technologies; Grand Island, NY) and GeneAmp® PCR System 9700 (Life Technologies). NCBI primer designing tool (see Table 2 for primer sequences) was used to design each primer. Real-time-PCR was performed using Power SYBR Green Master Mix (Life Technologies) on 96 well plates using a Quantstudio 6 Flex (Thermo Scientific; Pittsburgh, PA). For all runs, GAPDH was used as the internal housekeeping gene (the forward and reverse sequences are given in Table 2). The RT-qPCR reaction for each sample was conducted in duplicates (technical replicate). Water and no-template controls were used as negative controls for each primer set. The RT-qPCR data were analyzed using the QuantStudio™ 6 Flex Real-Time PCR System (Life technologies). Cycle threshold (Ct) values were obtained using auto baseline and applied to all amplicons of the same primer set. Delta-CT values were calculated using excel sheets provided by the authors [84].

**Table 2:** List of forward and reverse primer sequences used for qPCR analysis

Primer Name	Sequence 5´–3´
*TP53*/fwd	GTGCACGTACTCTCCTCCCC
*TP53*/rev	CAAAGCTGTCCCGTCCCAGA
MDM2/fwd	CAGGCCAATGTGCAATACCA
MDM2/rev	CAGCGATGTGCCAGAGTCTT
Noxa/fwd	TGGAGTGCACCGGACATAAC
Noxa/rev	ACTTCCCTAGCTCCACGACT
GAPDH/fwd	AGACAGCCGCATCTTCTTGT
GAPDH/rev	ATCCGTTCACACCGACCTTC
P21/fwd	CAGATCTTCCGGAGCCAGG
P21/rev	GACGACACAGGTGAGGAAGG
PINK1/fwd	TTGCCCCACACCCTAACATC
PINK1/rev	AGGGACAGCCATCTGAGTCC
Parkin/fwd	GGTCGATTCTGACACCAGCA
Parkin/rev	GGTGGGTTTAACTGGACCTCT
ROSA WT/fwd	CTGGCTTCTGAGGACCG
ROSA WT/rev	CCGAAAATCTGTGGGAAGTC
ROSA MT/fwd	CGTGATCTGCAACTCCAGTC
ROSA MT/rev	AGGCAAATTTTGGTGTACGG
TXN1/fwd	GCACCCAAATGGGAGAGTC
TXN1/rev	ACCAAGAAGCGTTAGAACTGG
Puma/fwd	ATGCCTGCCTCACCTTCATCT
Puma/rev	AGCACAGGATTCACAGTCTGGA
LC3B/fwd	CACTCCCATCTCCGAAGTGTA
LC3B/rev	TGCGAGGCATAAACCATGTA

### Radiation injury mouse models

Mice were irradiated with 5 Gy sub lethal or 9.5 Gy lethal total body irradiation (TBI) using a 137 cesium gamma irradiator (JL Shepherd, Glendale CA, USA) at a dose of 416.1 cGy / min. Mice were irradiated on a rotating platform. For calculating the survival curve, twenty-four hours following irradiation the mice were injected via IP with rTXN1 (1.6 mg/kg) every other day for five doses while the control group was administered with phosphate-buffered saline (PBS). Animal survival was monitored daily up to 30 days.

EML cell lines were radiated with indicated radiation dose using 137 cesium gamma irradiator [85].

### Plating EML cell lines after irradiation

After irradiation, EML cells were grown in Corning petri dish (25cm^2^) in complete IMDM cultured medium (Corning) supplement with 20% FBS (GeminiBio), 200 ng/ml mSCF (Fischer) and 1% penicillin/streptomycin (Sigma). After 24 h the cells were harvested and used for other experiments [85].

### Competitive bone marrow transplant

Bone marrow cells (1 × 10^5^) harvested at day 10 from tamoxifen treated- TXN1^fl/fl^ (control) and ROSA-CreER-TXN1^fl/fl^ mice were mixed with competitor bone marrow cells (1 × 10^5^) harvested from CD45.1 C57bl/6 J and injected by intravenous route into lethally irradiated (9.5 Gy total body irradiation) CD45.1 C57bl/6 J. Peripheral blood CD45.2 cells derived from TXN1^fl/fl^ and ROSA-CreER-TXN1^fl/fl^ mice were measured at weeks 4, 8 and 12.

### Limiting dilution competitive bone marrow transplant and serial transplantations

SLAM+KSL cells were sorted by flow cytometry from the bone marrow cells harvested at day 10 from tamoxifen treated- TXN1^fl/fl^ (control) and ROSA-CreER-TXN1^fl/fl^ mice (CD45.2). Two cell doses (50 SLAM+KSL cells or 200 SLAM+KSL cells) were mixed with competitor bone marrow cells (1 x 10^5^) harvested from CD45.1 C57bl/6J and injected by IV route into lethally irradiated (9.5Gy total body irradiation) CD45.1 C57bl/6J. Peripheral blood CD45.2 cells derived from TXN1^fl/fl^ and ROSA-Cre-TXN1^fl/fl^ mice were measured at month 1, 6, 9 and 10. For serial transplants, the 1st transplant recipient mice were sacrificed at 10 months after transplant. Bone marrow cells were harvested and injected into second, lethally irradiated (9.5Gy total body irradiation) CD45.1 C57bl/6J mice (5 x 10^6^ cells per mouse). Peripheral blood CD45.2 cells derived from TXN1^fl/fl^ and ROSA-Cre-TXN1^fl/fl^ mice were measured at month 2, 3 and 4. The second transplant recipient mice were sacrificed at 4 months and bone marrow cells were harvested and injected iv into third lethally irradiated (9.5Gy total body irradiation) CD45.1 C57bl/6J mice (5 x 10^6^ cells per mouse). Peripheral blood CD45.2 cells in the tertiary transplant recipient mice were followed.

### Lentiviruses

HEK 293 cells were transfected using the calcium-phosphate method with lentiviral vector plasmids and packaging plasmids PMD2.G and psPAX2 (Addgene, plasmid# 12259 and 12260). After 48 h of transfection, the cell supernatant was collected and concentrated using Lenti-X concentrator per manufacturer’s instructions. For lentiviral transfection, cells were seeded on six well plate (Corning) and infected with lentivirus in the presence of polybrene (8ug/ml). Cells were selected with puramycin (Sigma) for 7 days.

### CRIPSR-Cas9 Knock out experiments

For generating P53 knock out cells using CRISPR-Cas9 technique, single gRNAs targeting TrP53 gene were cloned in pX330-U6-Chimeric_BB-CBh-hSpCas9 and lentiCRISPR v1 vectors (Addgene) as described previously [86–88]. sgRNA sequences as follow: CTCTCTACAGATGACTGCCA, GGACGATCTGTTGCTGCCCC, GACGTGCCCTGTGCAGTTGT, CTCCTCCCCAGCATCTTATC, CCCGGATAGTGGGAACCTTC. Cells were transfected with two PX330 containing two sgRNAs targeting TrP53 exons 2–8. For generating TXN1 Knockout, cells were transfected with two PX330 for exons 2–4, sgRNAs sequences were used as follow: CCGATTTCGTACTACGTACC, GACAAGCTTGTCTCCCGCGG, CAACAGGTGGGGGAGTTCTC, TTTGACCCTTTTTATAAAAC. For generating TXN2 Knock out, cells were transfected with two PX330 for exons 2–3, sgRNAs sequences were used as follow: CGACCATCTTCTCTAGCCG, CGCGGTCCTAGGATCTTGCA, CGTGCTAGCCATCAAGAACG. Cells were tested for the successful deletion by qPCR and used for further experiments [89].

### Construction of p53 promoter pGL3 luciferase reporter system

DNA was isolated from EML cells using the DNeasy kit (Qiagen, Germatown,USA). PCR reactions were performed to isolate the promoter regions of P53 using the forward primer CTTCACCTGGATCCTGTGTCTT and the reverse primer GGAAACAGAGGAGGAGACTTCA. The PCR product (1200 bp) containing NheI and XbaI restriction enzyme digestion sites were loaded onto an 1% agarose gel and purified from the gel with the DNA gel-band purification kit (Qiagen, 28,704). The P53 promoter was cloned into pGL3-promoter vector containing a firefly luciferase gene (Promega, GenBank accession number U47295) at NheI and XbaI. The P53 promoter-pGL firefly luciferase reporter was used for experiments as indicated.

### Chromatin immunoprecipitation (ChIP) assay

ChIP assays were performed according to the manufacturer’s protocol (#91,820; Cell Signaling Technology, Danvers, MA). Briefly, the cells were collected and cross-linked with 1% formaldehyde. After centrifugation, the resulting pellets were sonicated and the chromatin solution was precleared with 30 mL of CHiP-Grade protein G magnetic beads (#9006; Cell Signaling Technology). The soluble fraction was collected, and the chromatin beads were incubated with positive control histone H3 rabbit (#4620; Cell Signaling), normal rabbit IgG (#2729; Cell Signaling), anti-TXN1 (PA587014, Thermofischer), anti- p53 (Cat#ab131442), mono-anti Flag M2 (Cat# F1804) antibodies at 4 °C overnight. ChIP-enriched DNA was analyzed by quantitative PCR using the *TP53* promoter primers as follows: forward CTTCACCTGGATCCTGTGTCTT and the reverse primer GGAAACAGAGGAGGAGACTTCA The enrichment of specific genomic regions was assessed relative to the input DNA, followed by normalization to the respective control IgG values.

### Protein degradation analysis (CHX chase)

Protein degradation assays were based on the use of protein synthesis inhibitor cycloheximide (CHX). EML control cells and EML/TXN1 KO cells were treated with CHX (100 µg/ml). Cells were collected at various time point (2, 4, 6, 8,10, 12, 14 and 24 h) after CHX treatment, and whole cell lysate was prepared and analyzed for p53 expression by western blot analysis and the signal density was quantitated by Image J.

### Cell cycle analysis

For assessment of proliferative status of BM and spleen cells, mice received i.p. injections of BrdU (10 mg/kg of body weight). Six hours later, the mice received a second i.p. injection of BrdU (10 mg/kg of body weight). Two hours later, BM and spleen were harvested and the MACS magnetic cell separation system (Miltenyi Biotech) was used to enrich mouse Lin^−^ cells from whole bone marrow and spleen. The cells were stained using FITC BrdU Flow Kit (BD Pharmingen™) according to the manufacturer's instructions. Data are representative of three independently performed experiments. Cells were analyzed by flow cytometry to determine the cell cycle profile. The numbers are average values ± SD from 4–5 mice in each group.

### Statistical analysis

All the data are presented as the mean ± SD. Comparisons were performed either by the student in order to test for analysis of variance for continuous data or by log-rank test for survival data. All statistical analyses were performed using Star View software (All statistical tests were performed with Graph Pad Prism V8 software). Values less than 0.05 were considered significant. *P < 0.05; **P < 0.01; ***P < 0.001; ****P < 0.0001.

## Results

### Thioredoxin-1 is required for the maintenance of hematopoiesis in mice

TXN has 2 forms: TXN1 and TXN2. TXN1 is the predominant isoform and is localized in the cytosol while TXN2 is restricted to mitochondria [6]. To investigate the role of TXN1 (encoded by the Txn1 gene) in hematopoiesis, we first crossbred TXN1^fl/fl^ mice with vavCre transgenic mice. The vavCre transgene constitutively inactivates floxed genes specifically in hematopoietic and endothelial cells. Thus, vavCreTXN1^fl/fl^ mice are expected to have complete TXN1 deletion selectively in both embryonic hematopoietic and endothelial cells. With multiple breeding pairs and over 100 offspring, we were not able to obtain a single vavCreTXN1^fl/fl^ mouse; all of the offspring were vavCreTXN1^wt/wt^ or vavCreTXN1^fl/wt^. Although further studies are needed to define at what time of organogenesis the embryo is most sensitive to the deletion of TXN1, this data suggests that TXN1 is crucial for embryonic hematopoiesis.

**Fig. 1:**
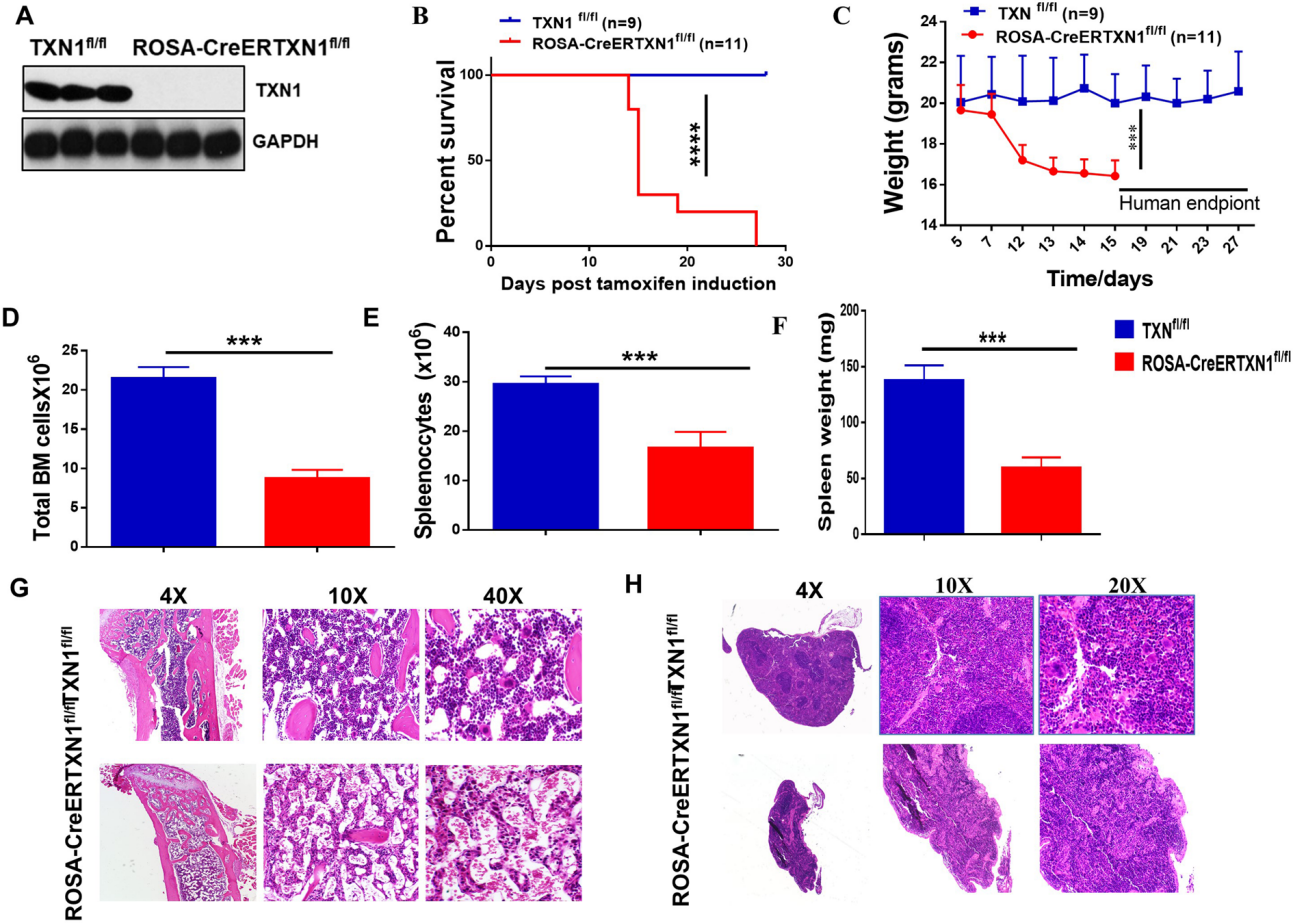
In-vivo thioredoxin deletion is lethal and is associated with reduced splenocytes and bone marrow cells in a murine model. **A** Mice with floxed TXN1 were crossed with mice carrying the ROSA-CreER transgene to develop an inducible model for thioredoxin deletion. TXN1^fl/fl^ (control) and ROSA-CreER-TXN1^fl/fl^ mice were given TAM (75 mg/kg) i.p. daily for 5 days. Mice were sacrificed at day 10. Thioredoxin was measured by Western blot in bone marrow cells. **B** Kaplan–Meier survival curve. TXN1^fl/fl^ mice and ROSA-Cre-TXN1^fl/fl^ mice were treated with TAM (75 mg/kg) i.p. daily for 5 days. Animal survival was monitored daily. **C** The weight of the mice as described in **B** was measured, TXN1 deletion resulted in drastic weight loss (humane endpoints required euthanasia following a > 20% reduction in body weight). **D** Total BM cells (one femur and one tibia) and total thymic cells following harvesting at day 10 of TAM injection. **E** Number of spleenocytes following harvesting at day 10 of TAM treatment. **F** Spleen weight (g) at day 10 of TAM treatment (n = 10 mice for each group each experiment, one of 3 separate experiments was shown). **G** Histology of bone marrow. Bone was harvested at day 10 from the mice as described in **F**. **H** Histology of spleen: Spleen was harvested at day 10 from the mice as described in **F**

To determine the role of TXN1 in adult hematopoiesis, we crossbred ROSA-CreER mice with TXN1^fl/fl^ mice and generated ROSA-CreERTXN1^fl/fl^ mice. The ROSA-CreER transgene enabled global, temporal deletion of floxed genes after tamoxifen (TAM) administration in vivo. TXN1^fl/fl^ mice (without ROSA-CreER) or ROSA-CreER mice (without TXN1^fl/fl^) were used as controls and we found there were no differences between TXN1^fl/fl^ mice or ROSA-CreER mice for use as controls. As a result, TXN1^fl/fl^ mice were used as controls in our subsequent experiments. We injected TAM (75 mg/kg i.p. daily for 5 days) to TXN1^fl/fl^ control mice or ROSA-CreERTXN1^fl/fl^ mice. TAM treatment for 5 days led to complete deletion of TXN1 in the bone marrow cells of ROSA-CreERTXN1^fl/fl^ mice harvested at day 10 (Fig. 1A and Additional file 1: Figure S1A). All ROSA-CreERTXN1^fl/fl^ mice treated with TAM died within 30 days (median survival was 15 days) accompanied by significant weight loss (Fig. 1B, C respectively, P < 0.001) compared to TXN1^fl/fl^ control mice treated with TAM. We harvested and examined major mouse organs (brain, heart, lung, liver, stomach, small intestine, large intestine, kidney, adrenal gland, bone marrow, and spleen) at day 10 of TAM treatment from TAM-treated TXN1^fl/fl^ control mice and TAM-treated ROSA-CreERTXN1^fl/fl^ mice (Additional file 1: Figure S1B, C). Histological evaluation revealed no changes in brain, heart, lung, liver, stomach, kidney, or adrenal gland after TXN1 deletion. There were disorganized intestinal villi and more inflammatory cell infiltration in small and large intestines of TAM-treated ROSA-CreERTXN1^fl/fl^ mice. The tissue/organ affected by TXN1 deletion most dramatically was hematopoietic, including bone marrow and spleen (Fig. 1D–H and Additional file 1: Figure S1B, C). Total BM cells (collected from 1 femur and 1 tibia per animal) were significantly reduced in TAM-treated ROSA-CreERTXN1^fl/fl^ mice compared to TAM-treated TXN1^fl/fl^ control mice (Fig. 1D). The spleen weight and the splenocyte numbers in TAM-treated ROSA-CreERTXN1^fl/fl^ mice were about one third the weight and one half the splenocyte number of TAM-treated TXN1^fl/fl^ control mice, respectively (Fig. 1E, F). Histological evaluation revealed a significant reduction of BM cellularity and absence of megakaryocytes in TAM-treated ROSA-CreERTXN1^fl/fl^ mice compared to the TAM-treated TXN1^fl/fl^ control group (Fig. 1G). Similarly, the cellularity of spleens in TAM-treated ROSA-CreERTXN1^fl/fl^ mice were significantly reduced and the structures were disorganized histologically (Fig. 1H). Failure in hematopoiesis likely plays a major role in the death of these mice.

**Fig. 2:**
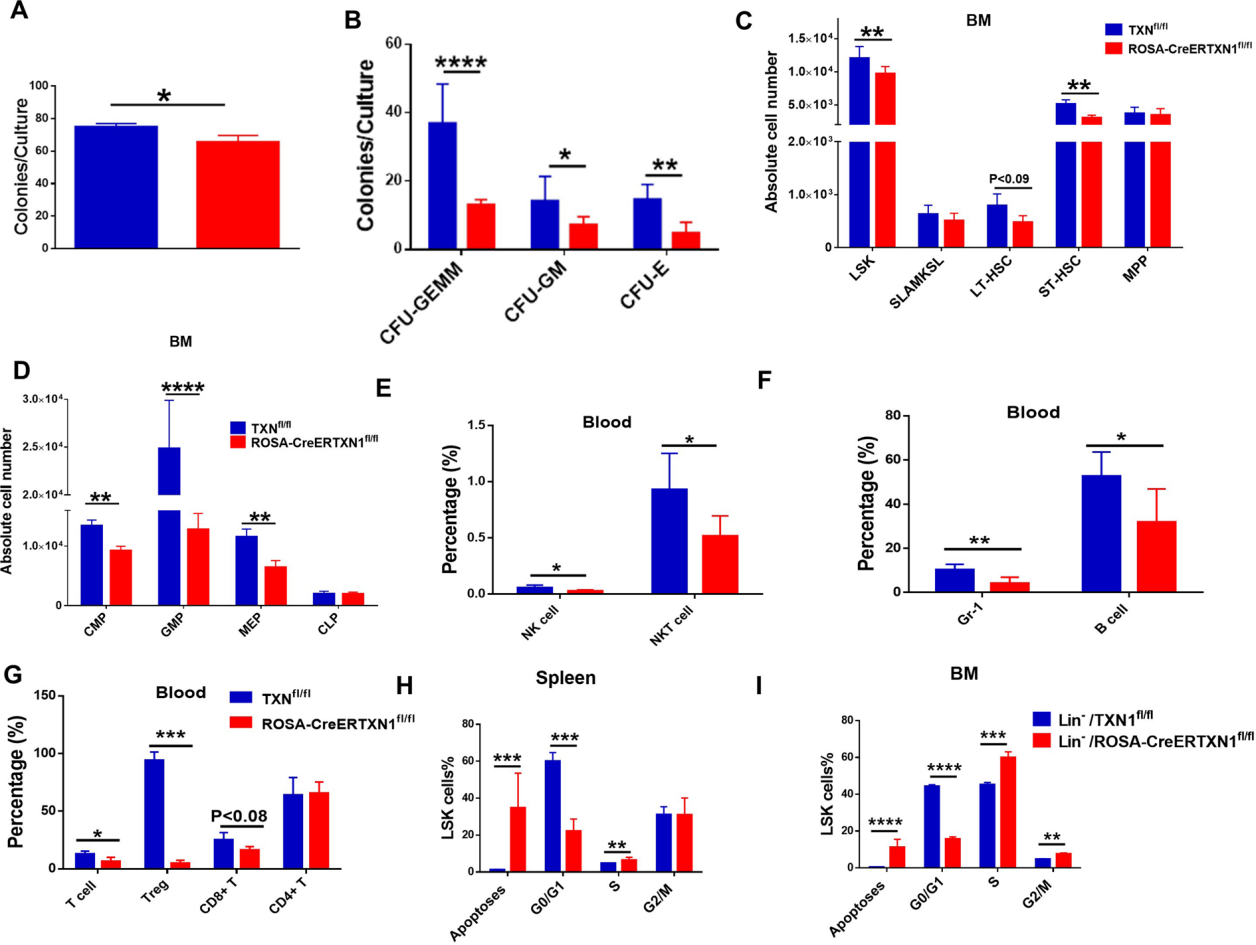
In-vivo thioredoxin deletion affects the self-renewal and differentiation of hematopoietic stem cells in a murine model. **A**, **B** Colony forming units. BM cells (2X10^4^) per dish were plated in Meth3434 semi-solid medium following harvesting at day 10 of TAM injection, and CFU were counted at day 12. Data represented mean ± s.d. **C**, **D** Various hematopoietic stem/progenitor cell subsets: TXN1^fl/fl^ (control) and ROSA-CreER-TXN1^fl/fl^ mice were treated with tamoxifen (75 mg/kg) i.p. daily for 5 days. Mice were sacrificed at day 10 and various hematopoietic stem cell populations (LSK, SLAMKSL, LT-HSC, ST-HSC, MPP, CMP, GMP, MEP and CLP) in the bone marrow were quantitated. Total number of cell populations per a femur and a tibia were shown. **E**–**G** the differentiated of erythroid and myeloid derived from HSCs were characterized by flow cytometry. Data represented mean ± s.d, n = 7–8, * P < 0.05, **P < 0.01, ***P < 0.001). **H**, **I** LSK cells in mice were labeled with BrdU in vivo, followed by FACS analysis to assess the cell cycle profile. Data are representative of three independently performed experiments

To further determine the role of TXN1 in hematopoiesis, we measured colony-forming unit (CFU-s) in bone marrow of TAM-treated ROSA-CreERTXN1^fl/fl^ mice and TAM-treated TXN1^fl/fl^ control group. CFU-s are a useful indicator for hematopoiesis and provides a valid ex vivo method for assessing the function of HSPCs [38]. We found that the bone marrow CFU-s at day 10 post TAM treatment were significantly reduced in TAM-treated ROSA-CreERTXN1^fl/fl^ mice compared to those in TAM-treated TXN1^fl/fl^ control mice (p < 0.05) (Fig. 2A). This reduction was seen in CFU-granulocyte, erythrocyte, monocyte/macrophage, megakaryocyte (CFU-GEMM), CFU-GM and CFU-E (Fig. 2B). We measured Lin-Sca-1 + c-Kit + (LSK) cells, SLAM + Lin-Sca-1 + c-Kit + (SLAM + KSL) cells, long-term (LT-HSC) or short-term (ST-HSC), and various components of committed progenitor cells including multi-potent progenitor cells (MPP), common myeloid progenitor cells (CMP), granulocyte-monocyte progenitor cells (GMP), megakaryocyte-erythrocyte progenitor cells (MEP), and common lymphoid progenitor cells (CLP). Deletion of TXN1 resulted in significant reduction in the number of LSK cells, LT and ST HSCs and various sets of hematopoietic progenitor cells (Fig. 2C, D). TXN1 KO mice showed significantly lower levels of NK, myeloid, B, and T cells in peripheral blood (Fig. 2E–G). We also performed in vivo BrdU labeling and measured apoptotic cells and cell cycles of LSK cells in the BM and spleen (Fig. 2H, I, Additional file 2: Figure S2). TXN1 deletion significantly increased apoptotic cell death of LSK cells, reduced quiescent Go/G1 LSKs, and drove more LSK cells into cell cycling, a cause of HSC exhaustion (Fig. 2H, I).

**Fig. 3:**
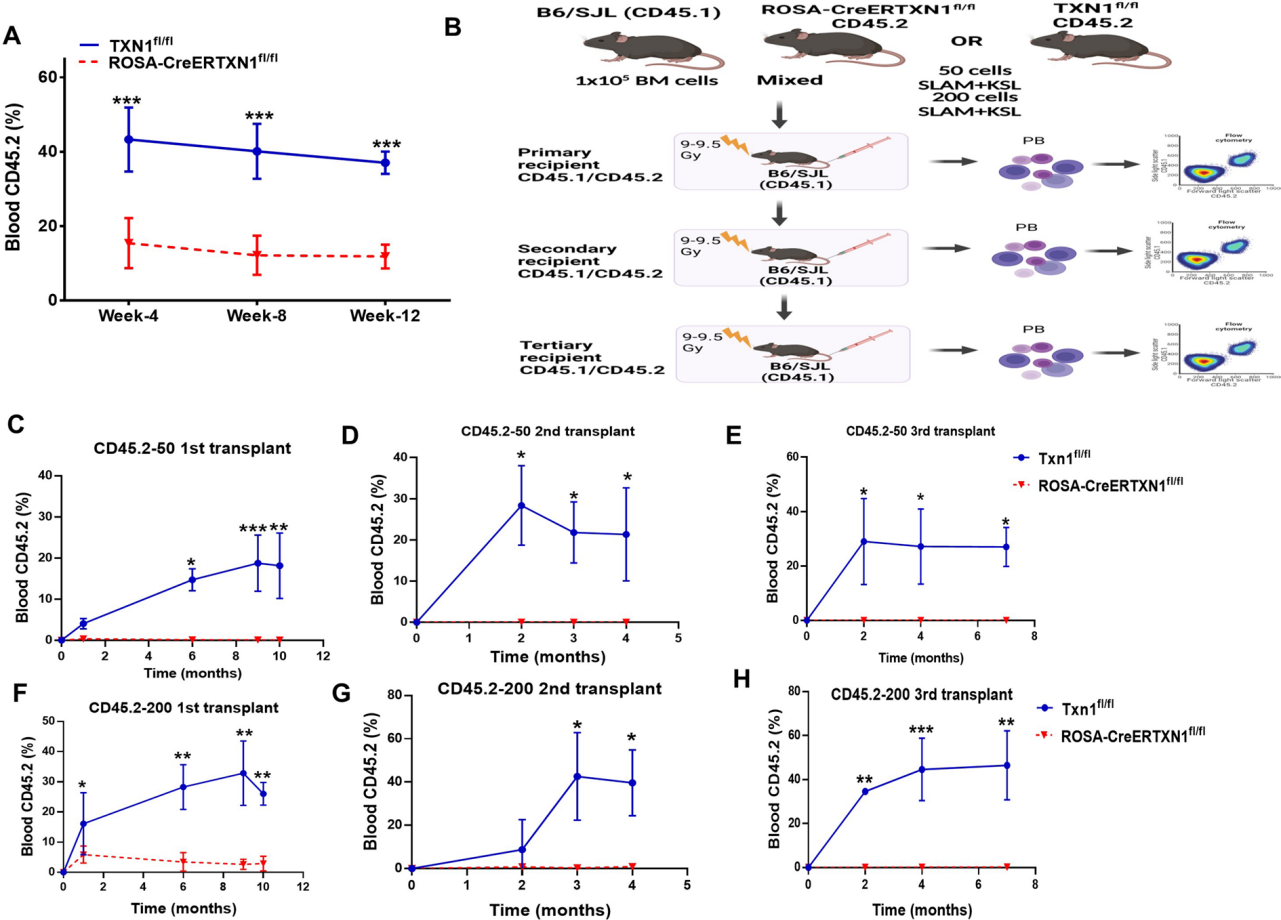
Thioreoxin deletion limits hematopoietic stem cells reconstitution ability in a murine model. **A** competitive transplantation: Bone marrow cells (1 × 10^5^) harvested at day 10 from TAM treated- TXN1^fl/fl^ (control) and ROSA-CreER-TXN1^fl/fl^ mice were mixed with competitor bone marrow cells (1 × 10^5^) harvested from CD45.1 C57bl/6 J and injected into lethally irradiated (9.5 Gy total body irradiation) CD45.1 C57bl/6 J. Peripheral blood CD45.2 cells derived from TXN1^fl/fl^ (control) and ROSA-CreER-TXN1^fl/fl^ mice were measured at indicated time-points (weeks 4, 8 and 12). **B** Schematic figure of limiting competitive bone marrow transplant: SLAM + KSL cells were sorted by flow cytometry from the bone marrow cells harvested at day 10 from TAM treated- TXN1^fl/fl^ (control) and ROSA-CreER-TXN1^fl/fl^ mice. Two cell doses (50 SLAM + KSL cells or 200 SLAM + KSL cells) were mixed with competitor bone marrow cells (1 × 10^5^) harvested from CD45.1 C57bl/6J and injected into lethally irradiated (9.5 Gy total body irradiation) CD45.1 C57bl/6J. **C**–**H** Peripheral blood CD45.2 cells derived from TXN1^fl/fl^ (control) and ROSA-Cre-TXN1^fl/fl^ mice were measured at indicated time-points in the first transplant recipient mice (month 1, 6, 9 and 10). The 1st transplant recipient mice were sacrificed at 10 months after transplant. Bone marrow cells were harvested and injected into second, lethally irradiated (9.5 Gy total body irradiation) CD45.1 C57bl/6J mice (5 × 10^6^ cells per mouse). Peripheral blood CD45.2 cells derived from TXN1^fl/fl^ (control) and ROSA-CreER-TXN1^fl/fl^ mice were measured at indicated time-points in the secondary transplant recipient mice (month 2, 3, 4). The second transplant recipient mice were sacrificed at 4 months and bone marrow cells were harvested and injected iv into third lethally irradiated (9.5 Gy total body irradiation) CD45.1 C57bl/6J mice (5 × 10^6^ cells per mouse). Peripheral blood CD45.2 cells in the tertiary transplant recipient mice were followed. ^**^p < 0.01; ^***^p < 0.001

We next investigated whether TXN1 KO could impact the ability of HSPCs to reconstitute the hematopoietic compartment of lethally irradiated recipient mice. We conducted a competitive bone marrow transplant. B6/SJL (CD45.1) mice were lethally irradiated (9.0–9.5 Gy) and transplanted with a mixture of 1 × 10^5^ bone marrow cells from B6/SJL (CD45.1) and 1 × 10^5^ bone marrow cells harvested from either TAM-treated ROSA-CreERTXN1^fl/fl^ mice or TAM-treated TXN1^fl/fl^ control mice (CD45.2). Peripheral blood CD45.2 (donor) and CD45.1 (background) was assessed by flow cytometry at 4, 8, and 12 weeks post-transplant. TXN1 deletion in HSPCs significantly impaired their reconstitution capacity (p < 0.001) (Fig. 3A). To further determine the effects of TXN1 on the self-renewal and long-term repopulating capacity of HSCs, we performed limiting dilution competitive (LDC) transplantation assay [39, 40]. We sorted SLAM + KSL cells from either TAM-treated ROSA-CreERTXN1^fl/fl^ mice or TAM-treated TXN1^fl/fl^ mice (CD45.2). We then mixed the sorted cells at doses of 50 and 200 cells with 1 × 10^5^ competitor bone marrow cells from B6/SJL (CD45.1) and injected these cells into lethally irradiated B6/SJL (CD45.1) mice (Fig. 3B). The frequencies of donor CD45.2 cell engraftment were measured at different time points (Fig. 3C–H, Additional file 1: Figure S3A, B and Additional file 4: Figure S4A-B). Secondary and tertiary transplants were also performed. At both 50 and 200 HSC cell doses, the levels of peripheral blood CD45.2 cells in primary, secondary and tertiary transplant recipient mice were dramatically lower in transplant mice receiving HSCs isolated from TAM-treated ROSA-CreERTXN1^fl/fl^ mice compared to those receiving HSCs isolated from TAM-treated TXN1^fl/fl^ mice (Fig. 3C–H). Additionally, the numbers of donor derived KSL cells in the bone marrow were significantly reduced in transplant recipients of HSCs isolated from TAM-treated ROSA-CreERTXN1^fl/fl^ mice compared to those receiving HSCs isolated from TAM-treated TXN1^fl/fl^ mice (Additional file 3: Figure S3A, B). These data demonstrated that the self-renewal and long-term repopulating capacity of HSCs were significantly impaired in TXN1 KO mice.

### Thioredoxin-1 deletion causes mitochondrial and biological dysfunctions in murine HSPCs

**Fig. 4:**
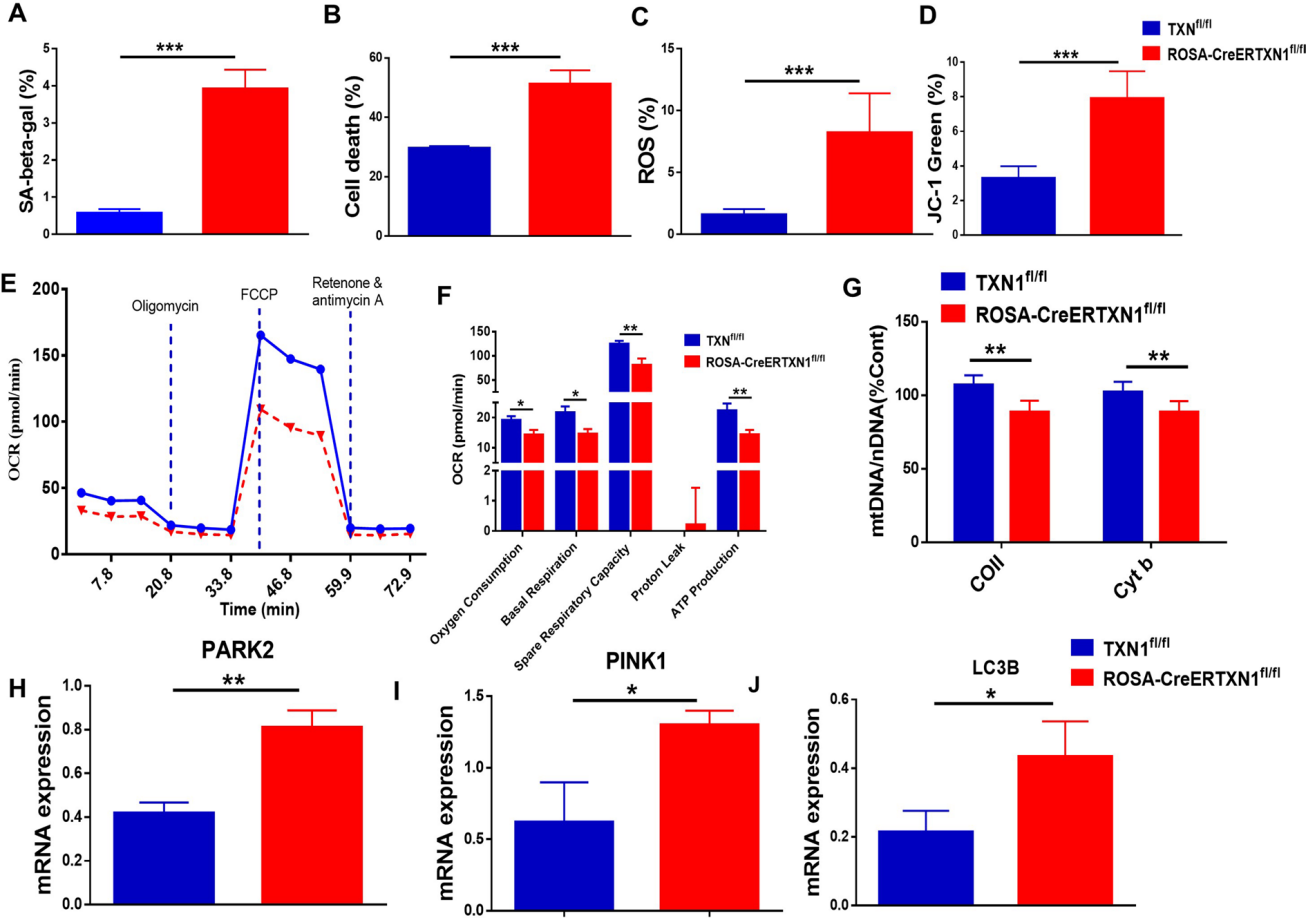
Biological effects of thioredoxin deletion on murine hematopoietic stem/progenitor cells. TXN1^fl/fl^ (control) and ROSA-CreER-TXN1.^fl/fl^ mice were treated with tamoxifen (75 mg/kg) i.p. daily for 5 days. Mice were sacrificed at day 10. Bone marrow cells were harvested and enriched for hematopoietic stem/progenitor cells using lineage depletion kit. The hematopoietic stem/progenitor- enriched cells were then measured for (**A**) beta-gal expression by flow cytometry staining, and Annexin (**B**). ROS measurement by carboxy-H2DCFDA staining. **C** JC probe to measure mitochondrial membrane polarization (**D**). **E**, **F** Seahorse measurement of oxygen consumption and cellular ATP concentration. **G** Relative mitochondrial DNA content (mtDNA:nDNA), The results were normalized to the ratio of mtDNA:nDNA of control cells. (n = 10 Mean ± SEM or SD). **H**–**J** PARK2, PINK1 and LC3B mRNA expression in murine HSPCs were quantified using qPCR (n = 3–5 Mean ± SEM or SD)

We further determined the effects of TXN1 deletion on cell survival and senescence of HSPCs. We harvested bone marrow cells at day 10 post TAM injection from TXN1^fl/fl^ control mice or ROSA-CreERTXN1^fl/fl^ mice and enriched for Lin- HSPCs. HSPCs from TAM-treated ROSA-CreERTXN1^fl/fl^ mice exhibited increased cell senescence, which was demonstrated by significant increases in SA-beta-galactosidase staining (Fig. 4A, p < 0.01) accompanied with increased cell apoptosis measured using Annexin V staining (Fig. 4B, p < 0.001). Consistent with the major role of TXN1 in redox homeostasis, HSPCs from TAM treated-ROSA-CreERTXN1^fl/fl^ conditional knockout mice showed elevated cellular ROS which was measured by Carboxy-H2DCFDA oxidation (Fig. 4C, p < 0.05).

It has been proposed that mitochondrial health and quality govern cell fate and differentiation of HSPCs [41]. Previously we have reported that TXN1 plays an important role in regulating mitophagy in myeloma cancer cells [42]. To study whether TXN1 deletion perturbs mitochondrial functions, we first measured JC-1 in Lin- HSPCs. JC-1 is a mitochondrial membrane potential-sensitive fluorescent dye used to detect the integrity and permeability of mitochondria [43]. TXN1 deletion led to depolarized mitochondrial membrane in Lin- HSPCs (Fig. 4D, p < 0.01). Seahorse XF assay was performed to assess mitochondrial mass and oxygen consumption [44]. Deletion of TXN1 impaired mitochondrial respiration either under basal conditions or after FCCP treatment (Fig. 4E). Net oxygen consumption, basal respiration rate, spare respiratory capacity, and ATP production were noticeably reduced in Lin- HSPCs from ROSA-CreERTXN1^fl/fl^ conditional knockout mice (Fig. 4F). Furthermore, TXN1 deletion resulted in a decrease in the ratio of mitochondrial to nuclear DNA (mtDNA:nDNA) (Fig. 4G) [45]. PTEN-induced putative kinase 1 (PINK1) and E3 ubiquitin-protein ligase Parkin (PARK2) are the master regulators of mitochondrial autophagy. Deletion of TXN1 in HSPCs up-regulated the mRNA expression of PINK1, PARK2, and LC3 (Fig. 4H–J respectively, P < 0.001). These data suggested that TXN1 affects the mitochondrial fitness either directly or indirectly through yet-to-defined mechanisms.

### Thioredoxin-1 deletion upregulates *TP53* signaling pathway in hematopoietic stem/progenitor cells in vivo and in vitro

**Fig. 5:**
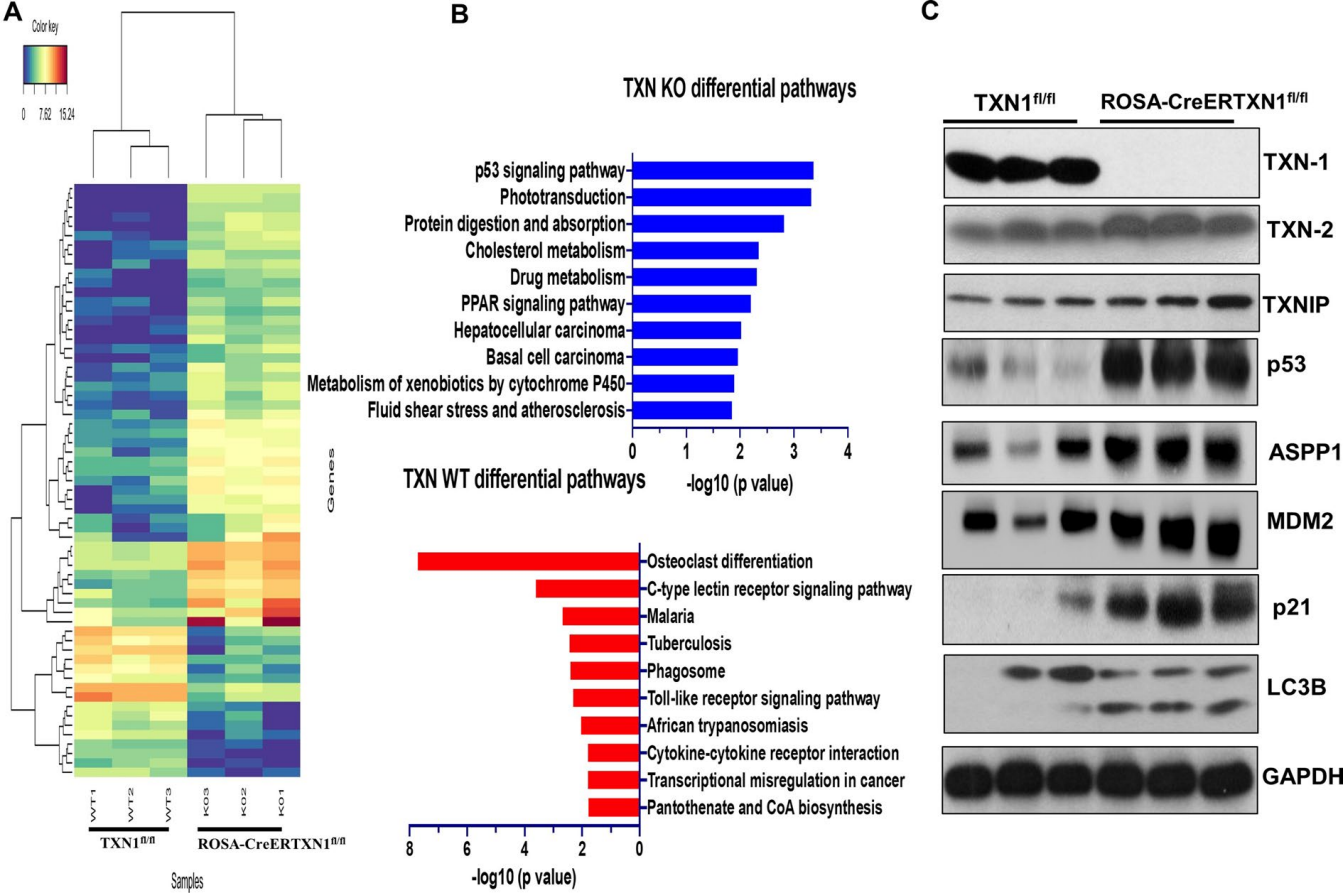
p53 signal pathway is significantly up-regulated in thioredoxin knockout Lin^−^ hematopoietic cells. TXN1^fl/fl^ (control) and ROSA-CreER-TXN1^fl/fl^ mice were treated with tamoxifen (75 mg/kg) i.p. daily for 5 days. Mice were sacrificed at day 10. Bone marrow cells were harvested and enriched for hematopoietic stem/progenitor cells using the lineage depletion kit. The hematopoietic stem/progenitor- enriched cells were then subject to Next generation RNA seq (**A**). **B** Pathway enrichment analysis. **C** Western blot analysis on bone marrow cells harvested from tamoxifen-treated TXN1^fl/fl^ (control) and ROSA-CreER-TXN1^fl/fl^ mice

To investigate the downstream molecular pathways of TXN1 deletion in murine HSPCs, we harvested bone marrow cells from TAM-treated ROSA-CreERTXN1^fl/fl^ and TAM-treated TXN1^fl/fl^ control at day 10 post TAM treatment and enriched bone marrow cells for Lin- HSPCs. We performed RNA sequencing (RNA-seq) on Lin- HSPCs from three mice in each group. Among the top differentially expressed gene sets (Fig. 5A, |GFOLD|> 1.5; false discovery rate [FDR] < 0.2, Additional file 5, Table S1) between Lin- HSPCs from TAM-treated ROSA-CreERTXN1^fl/fl^ and TAM-treated TXN1^fl/fl^ controls, the *TP53* signaling pathway was the most significant pathway up-regulated in TXN1 deleted HSPCs (Fig. 5B). Western blot analysis of Lin- HSPCs demonstrated specific deletion of TXN1, but not TXN2, nor thioredoxin interacting protein TXNIP; and up-regulated *TP53* as well as *TP53* canonical signaling pathway including ASPP1, MDM2, and p21 in HSPCs harvested from TAM-treated ROSA-CreERTXN1^fl/fl^ mice compared to HSPCs from TXN1^fl/fl^ control mice (Fig. 5C).

*TP53* is a key transcription factor that regulates the proliferation and expansion of HSCs [46–48]. *TP53* is implicated in HSCs quiescence and senescence and negatively regulates HSCs pool size. *TP53* deficiency increases HSPCs proliferation and the number of HSPCs [33]. To determine the role of *TP53* in mediating TXN1 effects on HSPCs, we performed ex vivo experiments using Erythroid myeloid lymphoid (EML) cell line. The EML cell line is an established multipotent hematopoietic precursor cell line that can be maintained in vitro in a medium including stem cell factor (SCF) [49]. We used CRISPR/Cas9 technique to delete TXN1 system in the EML cell line. Consistent with our in vivo conditional knockout mouse model, we found mRNA expression and protein level of *TP53* were upregulated after genetically deleting TXN1 (Additional file 6: Figure S5A–C). Additionally, MDM2, PUMA and NOXA genes and protein expressions were significantly increased in EML/TXN1KO compared to wild-type EML cells or EML cells transduced with Cas9 Control (Additional file 6: Figure S5A–C). Furthermore, similar to what we found in the TAM-treated ROSA-CreERTXN1^fl/fl^ mice, TXN1 knockout (KO) ex vivo led to reduced cell proliferation as measured by BrdU positive cells, increased cell senescence, higher percentage of apoptotic cells, and significantly reduced colony forming units (Additional file 6: Figure S5D-G).

As shown in Fig. 5C, the level of TXN2 protein noticeably increased after deletion of TXN1 in Lin- HSCs. To test whether the compensatory overexpression TXN2 has any effects on p53 and p53 canonical molecular pathway, we used CRISPR KO technique to delete TXN2 in EML cell line. Unlike TXN1 KO, deletion of TXN2 did not affect p53 level nor p53 canonical pathway (MDM2, Noxa or PUMA) (Additional file 7: Figure S6A). Similar to TXN1, deletion of TXN2 inhibited EML cell proliferation and increased cell senescence, mitochondrial depolarization and cell death (Additional file 7: Figure S6B–E). These data demonstrated that TXN1 deletion specifically up-regulated *TP53* expression in HSPCs.

### *TP53* plays a critical role in thioredoxin-1 mediated regulation of hematopoietic stem/progenitor cells

**Fig. 6:**
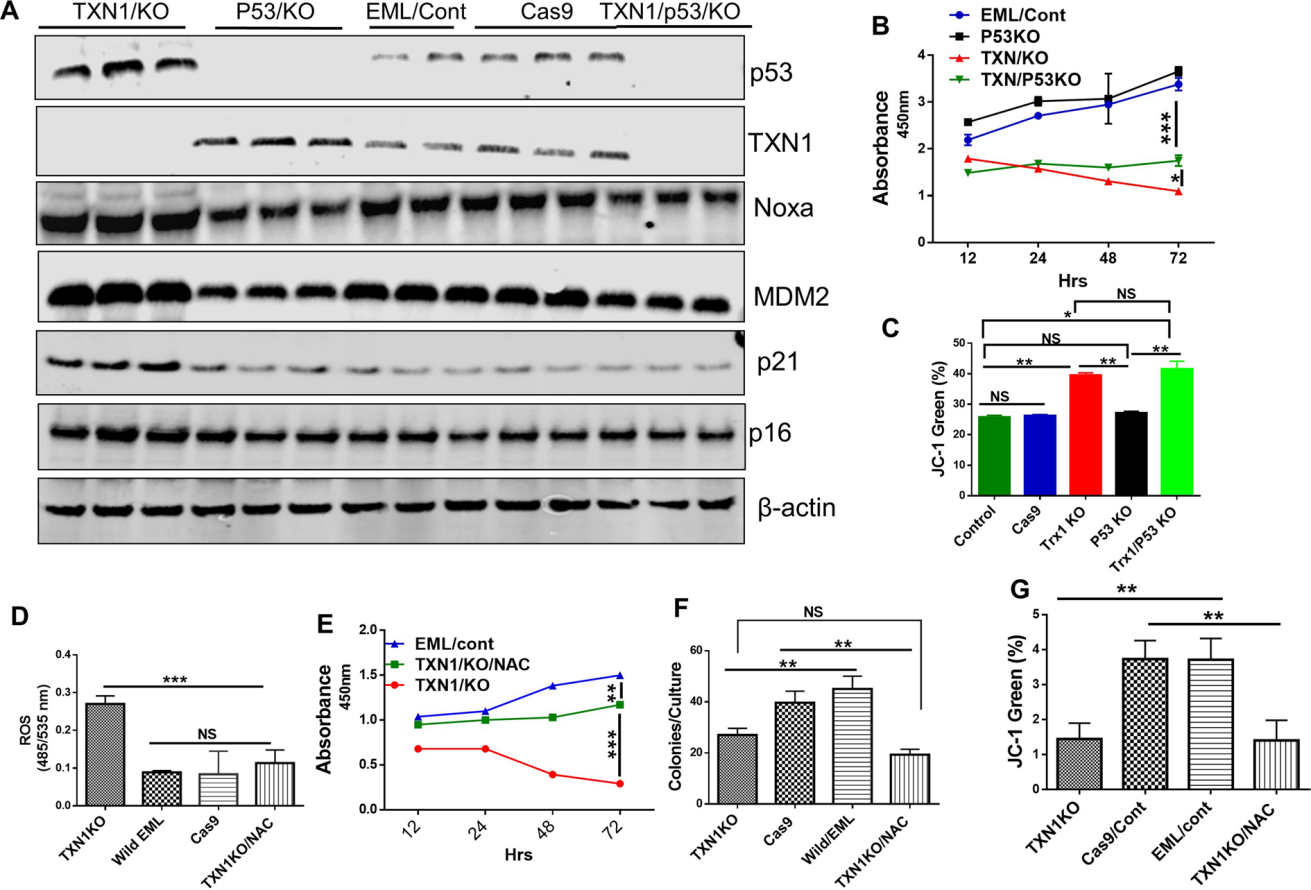
p53 plays a critical role in TXN1 mediated effects. **A** EML cells were transduced with Cas9 control, TXN1 specific CRISPR/Cas9, p53 specific CRISPR/cas9 or both. p53 and P53 downstream signaling pathway were assessed by immunoblot. β-actin was used to assess equal loading. **B** Cells proliferation rate was assessed using BrdU proliferation assay. **C** JC-I mitochondrial membrane depolarization was measured **D** ROS measurement by carboxy-H2DCFDA staining. EML/TXN1KO cells were treated with 250uM NAC and ROS levels were measured. **E** Cell proliferation. EML/TXN1KO cells were treated with 250uM NAC and cells proliferation rate was assessed using BrdU proliferation assay as described. **F** In vitro cultured colony forming unit in EML control cells, EML Cas9 control cells, EML TXN1 KO cells and EML TXN1 KO/NAC cells. EML/TXN1KO cells were treated with 250uM NAC, and plated at 2X10^4^/dish in Meth3434 semi-solid medium and incubated for 10-12 days. **G** JC-1 mitochondrial membrane depolarization was not affected by NAC treatment. EML/TXN1KO cells were treated with 250uM NAC and JC-1 levels were measured. (n = 4 Mean ± SEM or SD)

We next determined the role of *TP53* in TXN1-mediated effects on HSPC function and signaling pathways. To this end, we knocked out *TP53*, TXN1, or both using CRISPR/Cas9 technique in EML cells. TXN1 deletion significantly up-regulated Noxa, MDM2, p21 and p16 proteins expression. *TP53* knockout abrogated the effects of TXN1 deletion on Noxa, MDM2, p21 and p16 proteins expression (Fig. 6A). In addition, *TP53* knockout at least partially counteracted against TXN1 deletion and restored cell proliferation and viability that were significantly reduced by TXN1 KO to the baseline levels (Fig. 6B). Moreover, *TP53* knockout rescued EML cells from TXN1 deletion-induced mitochondrial membrane depolarization (Fig. 6C). These results indicated that *TP53* plays an important role in TXN1 mediated signaling pathways.

TXN1 can act as an antioxidant and functions independent of ROS. To determine if TXN1 regulates HSPCs through ROS-dependent or ROS-independent pathway, we knocked out TXN1 with CRISPR/Cas9 and then treated with 250 μM N-acetyl-L-cysteine (NAC). Although treatment with 250 μM of NAC significantly reduced the level of ROS and improved cell proliferation in EML/TXN1 KO cells (Fig. 6D, E), NAC treatment was unable to restore TXN1 deletion induced mitochondrial membrane depolarization nor to recover the colonies culture units (Fig. 6F, G respectively). Additionally, NAC failed to normalize the level of P53, MDM2, PUMA and Noxa after TXN1 deletion (Additional file 8: Figure S7A–E). In contrast, when we treated EML/TXN1KO with 5 μg/ml of recombinant thioredoxin1 (rTXN1) for 24 h [30], p53 and its downstream molecules such as MDM2, PUMA and Noxa were reduced noticeably (Additional file 9: Figure S8A–E). These results indicated that TXN1 regulates *TP53* canonical signaling pathway likely not due to increased ROS level but rather via direct effect of TXN1.

### Thioredoxin-1 regulates *TP53* expression at both post transcription and transcription levels.

**Fig. 7:**
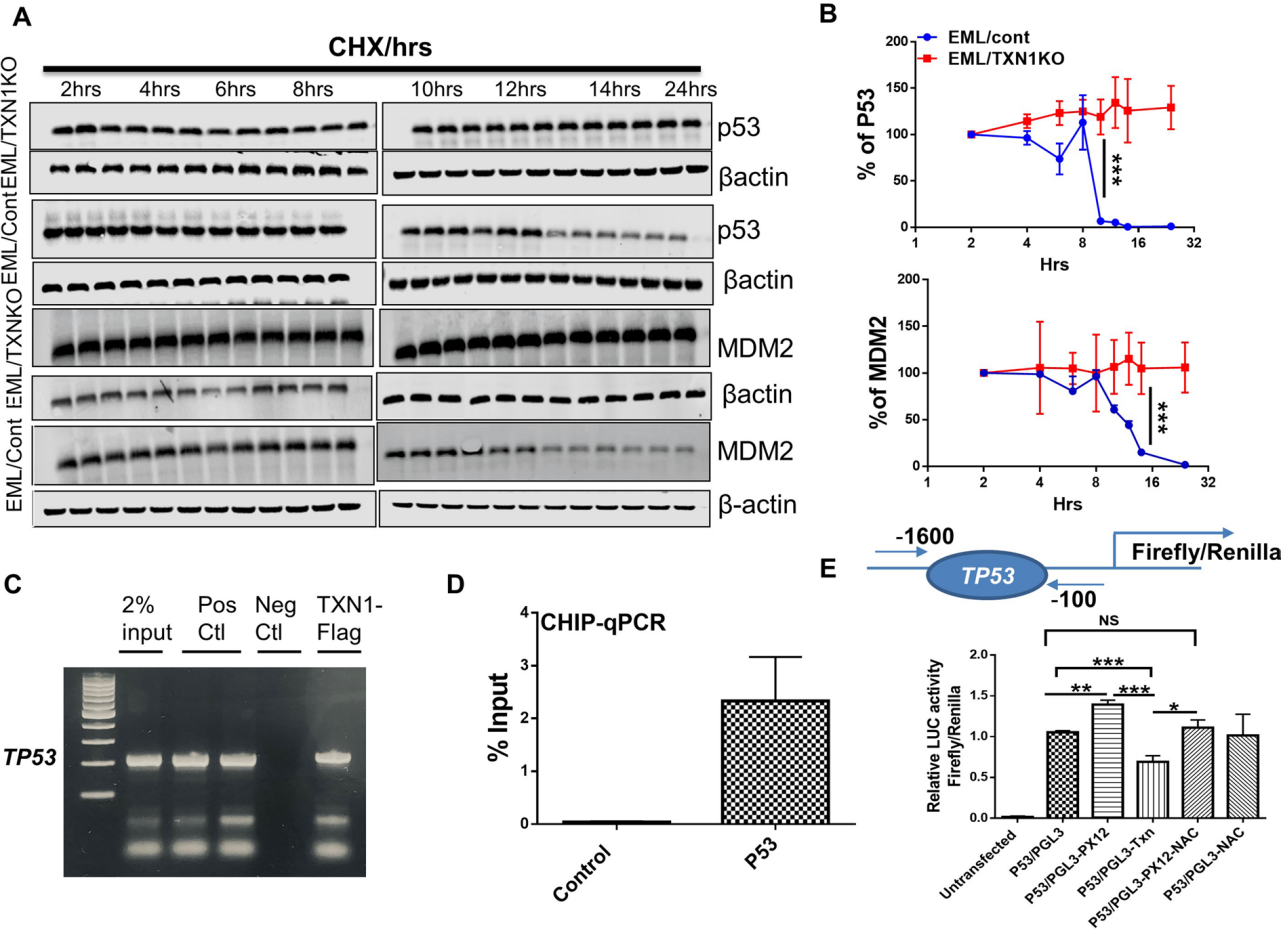
TXN1 regulates P53 at both post transcriptional and transcriptional levels. **A** TXN1 deletion prevented p53 and MDM2 from degradation. EML/TXN1KO and EML control cells were incubated with 50uM cycloheximide (CHX). At various time-points (2, 4, 6, 8, 10, 12, 14 and 24 h), the cells were harvested and p53, MDM2 and β-actin were measured by Western Blot analysis. **B** Expression levels of p53 and MDM2 were determined by image J software of the immunoblots in **A** (errors bars indicate mean ± sd; n = 3). **C** EML cells were transfected with flag-tag plasmid encoding TXN1 as indicated. Then the binding of TXN1 to the P53 promotor was analyzed by chromatin immunoprecipitation (CHIP-qPCR). **D** CHIP-qPCR representing the significant enrichment of the total input of the *TP53* promotor region in a CHIP experiment using the TXN1 flag-tag antibody relative to the enrichment by nonspecific IgG. **E**) *Tp53* promoter region (− 1600 to − 100) was cloned into pGL3 firefly/renilla luciferase reporter system. The p53 promoter-pGL3 reporter was transduced into EML cells and the EML cells were then treated with PX12 (thioredoxin inhibitor, recombinant TXN1 or NAC). Relative luciferase activity Luc (fold change) was calculated from the ratio of P53PGL3 Luc activity after normalization to the pRenilla

We next determined the mechanisms through which thioredoxin regulates *TP53* expression. The level of protein expression is controlled generally by either gene transcription and/or protein degradation. MDM2 binds directly to *TP53* and mediates P53 protein degradation. We first determined if TXN1 deletion affects the stability of p53 and MDM2. To this end, we treated TXN1 KO EML cells and Cas9 controlled EML cells with cycloheximide (CHX) to inhibit de novo protein synthesis and then measured P53 and MDM2 protein levels at different time points using immunoblot assays [50]. In EML cells transduced with Cas9 control vector, the protein level of p53 and MDM2 showed more than 80% reduction after 14 h of CHX treatment. In contrast, both p53 protein and MDM2 protein remained stable in TXN1 KO EML cells, and there is no reduction in p53 and MDM2 levels even 24 h after the CHX treatment (Fig. 7A, B). These data suggested that TXN1 deletion could protect p53 and MDM2 from protein degradation.

We found that TXN1 deletion increased both protein and mRNA expression of *TP53*, indicating that TXN1 could also regulate *TP53* expression at transcriptional level. To determine if TXN1 affects *TP53* transcription, we first performed chromatin immunoprecipitation (CHIP) followed by RT-qPCR. We found that TXN1 interacts with the promotor region of *TP53* (Fig. 7C, D). Then we cloned the *TP53* promoter/regulator region (− 1600 bp to 100 bp) into PGL3 firefly/renilla reporter system and transduced the PGL3-p53-reporter plasmid into EML cells. As shown in (Fig. 7E), treatment with PX12 (TXN1 inhibitor) increased *TP53* transcription while rTXN1 noticeably suppressed *TP53* transcription. Treatment with NAC did not significantly inhibit *TP53* transcription, further indicating that TXN1 regulates *TP53* transcription independent of ROS.

### Thioredoxin-1 is essential for maintaining and protecting HSPC functions during radiation exposure

HSCs must quickly respond to the stimuli such as viral infections, chemotherapy or irradiation to increase their expansion and differentiation in order to replenish immune and blood systems without stem cell pool depletion [51, 52]. We used radiation as a stressor to explore whether TXN1 could enhance the potency of HSPCs in response to the injury. To test the role of TXN1 in HSPC radioprotection, we irradiated EML cells (EML/IR) and EML/TXN1KO (EML/TXN1KO/IR) with 5 Gy, and then treated the cells with 10 μg/ml rTXN for 72 h. Radiation exposure activated P53 and P53 downstream pathway constituents (MDM2, Puma, and Noxa) and treatment with rTXN1 downregulated radiation-induced p53 pathway activation (Additional file 10: Figure S9A-B). EML cells were sensitive to the radiation injury and deletion of TXN1 rendered EML cells more sensitive to radiation. Treatment with rTXN1 protected EML control cells and EML/TXN1KO cells from radiation (Additional file 10: Figure S9C). TXN1 KO significantly prolonged p53 activation after radiation (Additional file 10: Figure S9D).

**Fig. 8:**
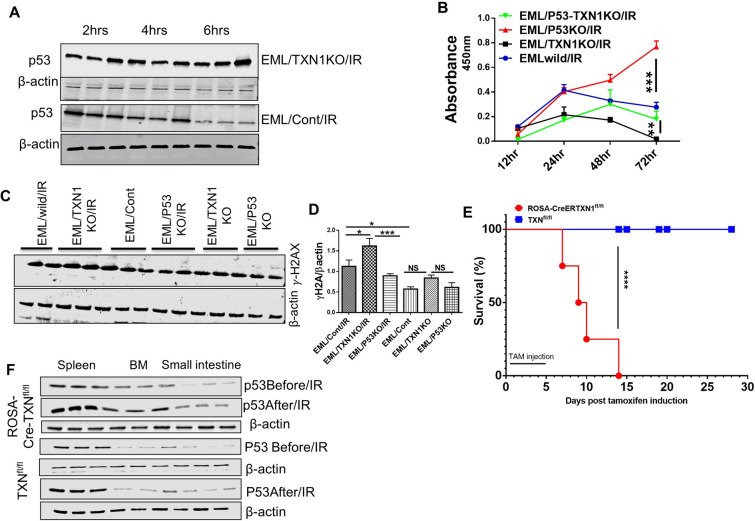
deletion of TXN1 attenuates HSPCs response to the stressor and reduces the survival of mice following sub-lethal dose of TBI. **A** P53 protein expression over time in response to 5 Gy radiation in EML/Cont and EML/TXN1KO cells. **B** BrdU cell proliferation. EML cells were transduced with Cas9 control, TXN1 specific CRISPR/Cas9, p53 specific CRISPR/Cas9 or both, and irradiated with 5 Gy. Cell proliferation was measured by BrdU incorporation. Data was plotted as mean ± SEM (n = 3, Data represents *p value < 0.01, **p value < 0.001).). **C** γ-H2AX expression in response to 5 Gy radiation in EML cell lines before and after deletion of TXN1 and/or TP53 genes. **D** Quantification of γ-H2AX expression using ImageJ software and normalized with βactin levels. (Data represent mean ± SD; n = 3). **E** Kaplan–Meier survival curve. TXN1^fl/fl^ mice and ROSA-CreER-TXN1^fl/fl^ mice were treated with TAM (75 mg/kg) i.p. daily for 5 days. Then the animals were exposed to 5 Gy radiation at day 7. Animal survival was monitored daily. **F** Western blot analyses of P53 protein expression in lysates prepared from (spleen, small intestine and bone marrow whole cells) from TXN1^fl/fl^ mice and ROSA-Cre-TXN1^fl/fl^ mice before and after 24 h of 5 Gy radiation exposure

In EML control cells, p53 increased at 4 h after irradiation but returned to baseline level by 6 h. In contrast, p53 expression in EML/TXN1KO cells remained elevated even at 6 h after radiation (Fig. 8A). To determine the role of p53 in TXN1 mediated radioprotection, we irradiated EML/TXN1KO, EML/p53KO, EML/TXN1-p53KO, and control EML cells. EML/p53KO cells were less sensitive to radiation and deletion of *TP53* rescued EML/TXN1KO cells from radiation induced growth inhibition at 72 h after radiation exposure (Fig. 8B). We also assessed γ-H2AX, a sensitive molecular marker of DNA damage and repair [53, 54]. Knocking out of *TP53* reduced γ-H2AX while TXN1 knockout increased γ-H2AX (Fig. 8C, D). These data support that TXN1 plays an important role in radioprotection in HSPCs through inhibiting *TP53* signaling pathway.

To assess the in vivo effects of TXN1 deletion on mouse survival following radiation, TAM-treated ROSA-CreERTXN1^fl/fl^ and TXN^fl/fl^ mice were exposed to a sub lethal dose of total body radiation (5 Gy IR). Mice lacking TXN1 were highly radiosensitive and died within a week after IR while TAM-treated TXN^fl/fl^ mice all survived (Fig. 8E). To determine whether TXN1 deletion affects *TP53* dynamics in vivo following irradiation, we measured the level of P53 protein in spleens, bone marrows and small intestines before and after radiation. p53 protein expression was up-regulated in BM, spleen and small intestine in TAM treated- ROSA-Cre-TXN^fl/fl^ mice compared to TAM-treated TXN^fl/fl^ mice at 24 h after radiation (Fig. 8F). The data demonstrates that TXN1 bestows a protective effect on HSPCs against radiation stressors.

## Discussion

HSCs reside in the hypoxic bone marrow niche environment at steady state and there are intrinsic and extrinsic factors that regulate HSCs’ self-renewal fate or, in certain situations, their ability to differentiate into mature blood and immune cells. Exhaustion stressors such as irradiation, viral infection and myelotoxic chemotherapeutic agents disturb the steady state of HSCs, which impairs their ability to maintain lifelong hematopoiesis [55, 56]. In this study, we explored the molecular mechanisms through which TXN1 regulates HSCs in steady state and protects HSCs from stressful injuries. Our initial finding that no viable Vav-Cre-TXN^fl/fl^ mouse were produced suggests that TXN1 is crucial for hematopoietic development in early embryogenesis. To determine the role of TXN1 in adult hematopoiesis and to further define the relevant molecular mechanism [57–59], we generated TXN1 conditional knockout mice (Rosa-CreERTXN1^fl/fl^ mice) that allows for induced deletion of TXN1 after TAM administration. Although disorganized villi and inflammatory cell infiltration were observed in small and large intestines, the major phenotypes of the mice lacking the TXN1 are characterized by the defects in hematopoiesis and the inability of HSCs to reconstitute hematopoiesis in primary, secondary and tertiary transplant recipient mice.

We found that deletion of TXN1 significantly reduced the number and function of HSPCs. The numbers of LT HSCs and ST HSCs cells in TXN1 knockout mice (TAM-treated ROSA-CreERTXN1^fl/fl^ mice) were strongly reduced compared to those in wild-type mice (TAM-treated TXN1^fl/fl^ mice), and the numbers of committed progenitor cells such as MPPs, CMPs, GMPs and MEPs) were also significantly reduced in TXN1 knockout mice. All lineages of peripheral blood cell types were reduced in TXN KO mice. The bone marrow sections from TAM-treated ROSA-CreERTXN1^fl/fl^ mice were hypocellular, indicative of hematopoietic cell depletion. Furthermore, using competitive transplant assay and limiting dilution serial transplantations, we unequivocally demonstrated that HSCs depleted of TXN1 were unable to self-renew or to differentiate into committed progenitor cells to reconstitute hematopoiesis in transplant recipient mice.

In the current study, we used ROSA-CreER transgene for conditional knockout of TXN1. Activation of ROSA-CreER transgene with TAM results in global deletion of TXN1 in all organs. We have examined major organs of the mice (brain, heart, lung, liver, stomach, small intestine, large intestine, kidney, adrenal gland, bone marrow, and spleen) and found that bone marrow and spleen are the predominant organs affected by TXN1 KO. We have recently generated Mx-1-Cre TXN1 conditional knockout mice, which leads to selective deletion of TXN1 in hematopoietic tissues/organs upon injection of pIpC injection. Mx-1-Cre TXN1 conditional knockout mice will allow us to further define the effects of TXN1 in hematopoiesis.

The causes of the death in the TXN1 KO mice are not completely characterized. Given the dramatic defects on hematopoiesis and no significant histopathological changes in other major organs, failure in hematopoiesis likely plays a major role in the death of these mice after TAM treatment. It is also possible that nutrition intakes due to dysfunction in small and large intestines could also play a role in the death of TXN1 KO Mice.

Recent studies reported that mitochondrial mass and mitochondrial membrane potential (MMP) are critical to HSPC multipotency and differentiation fate [41, 60, 61]. Interestingly our data revealed that mtDNA copy number, ATP content, and oxygen consumption changed significantly after deletion of TXN1 in HSPCs. These physiological functions were accompanied by the upregulation of Pink1, Park2 and LC3 mRNA expressions. Studies have implicated mitochondrial thioredoxin (encoded by TXN2) and thioredoxin inhibitory protein (TXNIP) in regulating mitochondrial function and fitness. It is possible that TXN1 may also possess direct or indirect effects on mitochondrial energy metabolism and mitophagy.

Gene ontology analysis of our data set showed that P53 signaling pathway was significantly upregulated in TXN1 null Lin- HSCs. Our results were further confirmed using EML cells as in vitro HSPC model. We found that knocking out TXN1 in EML cells dramatically upregulated P53 expression and P53 canonical pathway genes including PUMA, NOXA and MDM2. Moreover, TXN1 null Lin- HSCs and EML cells lacking TXN1 showed noticeable senescence and apoptotic signaling accompanied with significant reduction in colony forming units. P53 has been described as a “cellular gatekeeper” because of its central role in coordinating the cellular responses to a broad range of cellular stress factors. P53 also plays a major role in regulating HSC number and senescence through a variety of downstream target genes such as P21, PUMA, NOXA and P38 [33, 34, 62]. TXN1 is the major antioxidant protein in the cell, and its major role is to keep the reducing equivalent that sustains a variety of cell biological functions. One possible explanation for the effect of TXN1 deletion on p53 up-regulation is that deletion of TXN1 tilts the cells towards oxidation reactions, creating a state of oxidative stress. The inevitable outcome of oxidative stress is an accumulation of reactive oxygen species (ROS) that can cause DNA damage which then overexpresses P53 to arrest cell cycle and trigger apoptotic signaling pathways. Interestingly, supplementation of classical antioxidant NAC was found insufficient to restore the expression of p53 and p53 downstream pathways nor to recover cell proliferation and cell senescence in EML TXN1KO cells when compared to wild-type cells. In contrast, treatment with recombinant TXN1 restored the level of P53 expression, cells senescence, and cells proliferation. These data indicate that the defective proliferation and upregulation of P53 signal following TXN1 deletion is at least partially independent of cellular ROS. Thus, our data points to TXN1 as a direct regulator for P53 expression.

We investigated how TXN1 regulates p53 expression at both post transcription and transcription levels. The level and activity of p53 are largely regulated by posttranslational modifications such as phosphorylation, ubiquitination, and acetylation [63–66]. Over 35 different amino acids within p53 have been shown to be modified in various studies. The cellular p53 level is primarily controlled by the MDM2 or MDMX mediated ubiquitin-proteasomal degradation mechanism. MDM2 (mouse double minute 2 homolog) is an E3 ubiquitin ligase and forms an auto regulatory feedback loop with p53: while MDM2 can induce p53 degradation and inhibit p53 activity, p53 stimulates the expression of MDM2. MDM2 works with p21 to induce cell senescence and cell cycle arrest. ASPP1 synergizes with p53 to specifically transactivate proapoptotic genes including BAX, PUMA and PIG3 [67, 68]. Phosphorylation of p53 is the first crucial step of p53 stabilization. DNA damage-sensing kinases (ATM and ATR) and checkpoint kinases (Chk1 and Chk2) can phosphorylate p53 and induce p53 stabilization through dissociation from MDM2[69–71]. We have found that TXN1 deletion prevented both p53 and MDM2 from degradation. The exact mechanism through which TXN1 regulates the stability of p53 remains to be determined. Our unpublished data indicated recombinant TXN1 can down-regulate radiation-induced pATM and pChk1. It is possible that TXN1 regulates p53 stability through ATM/ATR and CHK1/2*.*

Interestingly, our CHIP-PCR experiment and pGL3-Firefly/renilla reporter system demonstrated that TXN1 affects P53 transcription. Previous studies showed that TXN1 could affect the DNA binding activity of several genes such as NFkB and NFAT via modification of the redox state of cysteine 62, or through modification of the disulfide bond between the two cysteine residues located on the class II HDACs and DnsJb5 to form a multiprotein complex which in turns regulates the nucleocytoplasmic activity of NFAT gene [72, 73]. More studies have found that TXN1 interacts with the tumor suppressor gene PTEN and inhibits PTEN lipid phosphatase activity in a redox dependent manner [74, 75]. More recently, it was shown that TXN1 binds to S100P gene promoter region and regulates S100P gene transcription in colon rectal cancer cell lines [76]. it is noteworthy that although there are many proteins reported to bind and regulate p53, very few have been shown to be physically recruited to the promoters of p53 [65, 66, 77, 78]. Additional studies are warranted to further understand how TXN1 interacts with P53 and inhibits p53 activity at transcription level. It is possible that TXN1 may regulate p53 transcription indirectly via other transcription factors such as an indirect interaction with the p53 promoter via protein–protein interactions with some other sequence-specific DNA binding protein.

We have previously shown that TXN1 protected HSPCs from radiation injury [30, 90]. Our current study not only extends our previous findings but also provides the detailed insight into the molecular mechanisms through which TXN1 regulates HSPCs function. Exposing EML cells to the sub lethal dose of irradiation upregulated P53 and P53 downstream genes, indicating that P53 plays an important role in mediating apoptosis in HSPCs in response to the radiation injury. Furthermore, we found that deletion of TXN1 in EML cells or using a conditional TXN1 knockout mice leads to an elevated radio-sensitivity. Administration of recombinant thioredoxin has comprehensive beneficial effects, not only helping to inhibit apoptosis and enhance cell proliferation, but also increase the survival of mice exposed to TBI. Such a profound beneficial effect of Thioredoxin in the context of radiation damage appears to be likely more attributed to better maintenance of biological functions in the exposed stem cells. These findings strongly justify TXN1 as an attractive molecule for developing therapeutic agents in mitigation against radiation-induced hematopoietic injury and for enhancing hematopoietic recovery after hematopoietic stem cell transplantation.

## Conclusions

Our study provided a new insight into the roles of TXN1 in HSC functions and the molecular mechanisms through which TXN1 regulates HSCs. Furthermore, our findings expand our knowledge base and suggest the utility of TXN1 in clinical applications such as for radiation protection and bone marrow transplant.

## Supplementary Information

**Additional file 1: Figure S1**. deletion of Thioredoxin leads to significant effects on different organs in mice. A) Schematic representation of tamoxifen injection in mice carrying the ROSA-Cre transgene to develop an inducible model for thioredoxin deletion. TXN1^fl/fl^ (control) and ROSA-CreER-TXN1^fl/fl^ mice were given TAM (75mg/kg) i.p. daily for 5 days. Mice were sacrificed at day 10. B) Representative photographs of spleens. TXN1^fl/fl^ (control) and ROSA-Cre-TXN1^fl/fl^ mice were treated with TAM (75mg/kg) i.p. daily for 5 days. Mice were sacrificed at day 10 and spleens were harvested. C) Histological sections (H&E staining) of the major organs of TXN1^fl/fl^ (control) and ROSA-Cre-TXN1^fl/fl^ mice, including Brain, heart, Lung, liver, stomach, small intestine, large intestine , kidney, and adrenal gland, No appreciable inflammatory response, cell degeneration, necrosis, or embolism was detected between TXN1^fl/fl^ (control) and ROSA-Cre-TXN1^fl/fl^ mice, Scale bar = 100 um.

**Additional file 2: Figure S2**. Conditional knockout of thioredoxin causes apoptosis and cell cycle activation of HSCs. LSK cells in mice were labeled with BrdU in vivo, followed by FACS analysis to assess the cell cycle profile, Brdu FITC and 7-AAD. Data in the two center panels were displayed as a histogram on the right. Data were representative of three independently performed experiments.

**Additional file 3: Figure S3**. Conditional knockout of thioredoxin leads to significant suppression of hematopoiesis. A-B) The 2nd transplant recipient mice were sacrificed at 4 months after transplant. Bone marrow cells were harvested and injected into third, lethally irradiated (9.5Gy total body irradiation) CD45.1 C57bl/6J mice (5 x 10^6^ cells per mouse). Peripheral blood CD45.2 cells derived from TXN1^fl/fl^ (control) and ROSA-CreER-TXN1^fl/fl^ mice were measured at the indicated time-points in the tertiary transplant recipient mice (months) (5 x 10^6^cells per mouse).

**Additional file 4: Figure 4**. Gating strategy of bone marrow hemopoietic stem cells of TXN1^fl/fl^(control) and ROSA-CreER-TXN1^fl/fl^ mice. The gating strategy was performed as follows: A) LSK cells: Lin- Scal^+^ C-Kit^+^; LT-HSC: Lin- Scal^+^ C-Kit^+^ CD150^+^ CD48^-^; ST-HSC: Lin^-^ Scal^+^ C-Kit^+^ CD150^-^ CD48^-^; MPP: Lin- Scal^+^ C-Kit^+^ CD150^-^ CD48^+^. B) MP: Lin^-^ Scal^-^ C-Kit^+^; GMP: Lin^-^ Scal^-^ C-Kit^+^ CD16/32^+^ CD34^+^; CMP: Lin^-^ Scal^-^ C-Kit^+^ CD16/32^-^ CD34^+^; MEP: Lin^-^ Scal^-^ C-Kit^+^ CD16/32^-^ CD34^-^; CLP: Lin^-^ Scal^+^ C-Kit^+^ CD127^+^.

**Additional file 5: Table S1**.

**Additional file 6: Figure S5**. TXN1 deletion up-regulates P53 signaling pathways and impairs the proliferation and survival of EML cells. A: p53 and p53 downstream pathways: EML cells were transduced with TXN1 specific CRISPR/cas9 or Cas9 control vector. P53, MDM2, PUMA and Noxa mRNA expression in EML cell lines were quantified using qPCR (n=3 Mean+/-SEM or SD). B: Western blot analyses of P53 and P53 family expression in EML cell lines after deletion of TXN. protein quantity was compared to β-actin protein as housekeeping protein. C: quantification of P53, MDM2, PUMA and Noxa protein expression in relative to β-actin housekeeping protein. D) Knocking out TXN1 inhibits EML cells proliferation as detected by BrdU cell proliferation assay. EML control cells, EML Cas9 control cells and EML TXN1 KO cells were seeded at 5000 cell/well in 96well plate and incubated at different time points. Then, 10 μM BrdU was added to the cells and incubated for 4 hr. (E-F) respectively represent beta-gal expression and Annexin by flow cytometry staining in EML control cells, EML Cas9 control cells and EML TXN1 KO cells. G) In vitro cultured colony forming unit granulocyte macrophage (CFU-GM) in EML control cells, EML Cas9 control cells and EML TXN1 KO cells. Cells (EML/TXN1KO, EML/Cas9control and EML/control) were plated at 2X104/dish in Meth3434 semi-solid medium and incubated for 10-12 days.

**Additional file 7: Figure S6**. TXN2 deletion impairs the proliferation and survival of EML cells. A) EML cells were transduced with TXN1 or TXN2 specific CRISPR/cas9 or Cas9 control vector. p53 and p53 family expression in EML cell lines after deletion of TXN1 or TXN2 were quantified using immunoblot, protein quantity was compared to β-actin protein as housekeeping protein. (n=3 Mean+/-SEM or SD). B) Knocking out TXN1 inhibits EML cells proliferation as detected by BrdU cell proliferation assay. EML control cells, EML Cas9 control cells, EML TXN1 KO and EML TXN2 KO cells were seeded at 5000 cell/well in 96well plate and incubated at different time points. Then, 10 μM BrdU was added to the cells and incubated for 4 hr. C-E) respectively represent beta-gal expression, JC-1 and Annexin by flow cytometry staining in EML control cells, EML Cas9 control cells, EML TXN2 cells and EML TXN1 KO cells.

**Additional file 8: Figure S7**. N-acetylcysteine supplement unable to normalize the expression level of P53 in EML cell lines. A) Representative western blots displaying P53 and P53 family member protein expressions in EML control, EML/TXN1KO and EML/TXN1KO treated with 250uM NAC. Beta-actin was used as a loading control. B-E) Western data was quantified using ImageJ software and normalized with beta-actin levels. Data represented mean±SD.

**Additional file 9: Figure S8**. rTXN1 supplement downregulated P53 protein expression in EML/TXN1KO cells. A) Representative western blots displaying P53 and P53 family member protein expressions in EML control, EML/TXN1KO and EML/TXN1KO treated with rTXN1 32ug/ml. Beta-actin was used as a loading control. B-E) Western data was quantified using ImageJ software and normalized with beta-actin levels. Data represented mean±SD.

**Additional file 10: Figure S9**. radiation enhanced apoptotic-signaling pathways in EML cells. A) Representative western blot analyses of P53 and P53 family genes expression in response to 5Gy radiation in EML cell lines and supplied with rTXN1 for 24hrs. B) Western data was quantified using ImageJ software and normalized with beta-actin levels. Data represented mean±SD. C) BrdU cell proliferation. EML control and EML TXN1 KO cells were irradiated with 5Gy, and plated in 96well plate/5000 cells/well and incubated with or without recombinant TXN1 for different time points as indicated in the figure. Plate was read at dual wavelength 450/550 nm. Data was plotted as mean ± SEM (n = 3). D) EML control and EML TXN1 KO cells were irradiated with 5Gy, and treated with or without recombinant TXN1. Cells lyses were collected after 24hrs of radiation and β-actin was used as internal housekeeping protein.

## Data Availability

The data that support the findings of this study are available on request from the corresponding author. RNA seq data has been attached to this manuscript.

## Abbreviations

HSCs: Hematopoietic stem cells
HSPCs: Hematopoietic stem/progenitor cells
CFUs: Colony forming units
TXN1: Thioredoxin-1
TXN2: Thioredoxin-2
TXNIP: Thioredoxin inhibitory protein
NADPH: Nicotinamide adenine dinucleotide phosphate
NAC: N-acetyl-L-cysteine
EML: Erythroid myeloid lymphoid
CHX: Cycloheximide
TBI: Total body irradiation
