# Supplementary figures and images for: Thioredoxin-1 regulates self-renewal and differentiation of murine hematopoietic stem cells through p53 tumor suppressor
Source: Exp Hematol Oncol. 2022 Oct 31;11:83. doi: 10.1186/s40164-022-00329-3 (PMC9624023; doi:10.1186/s40164-022-00329-3)

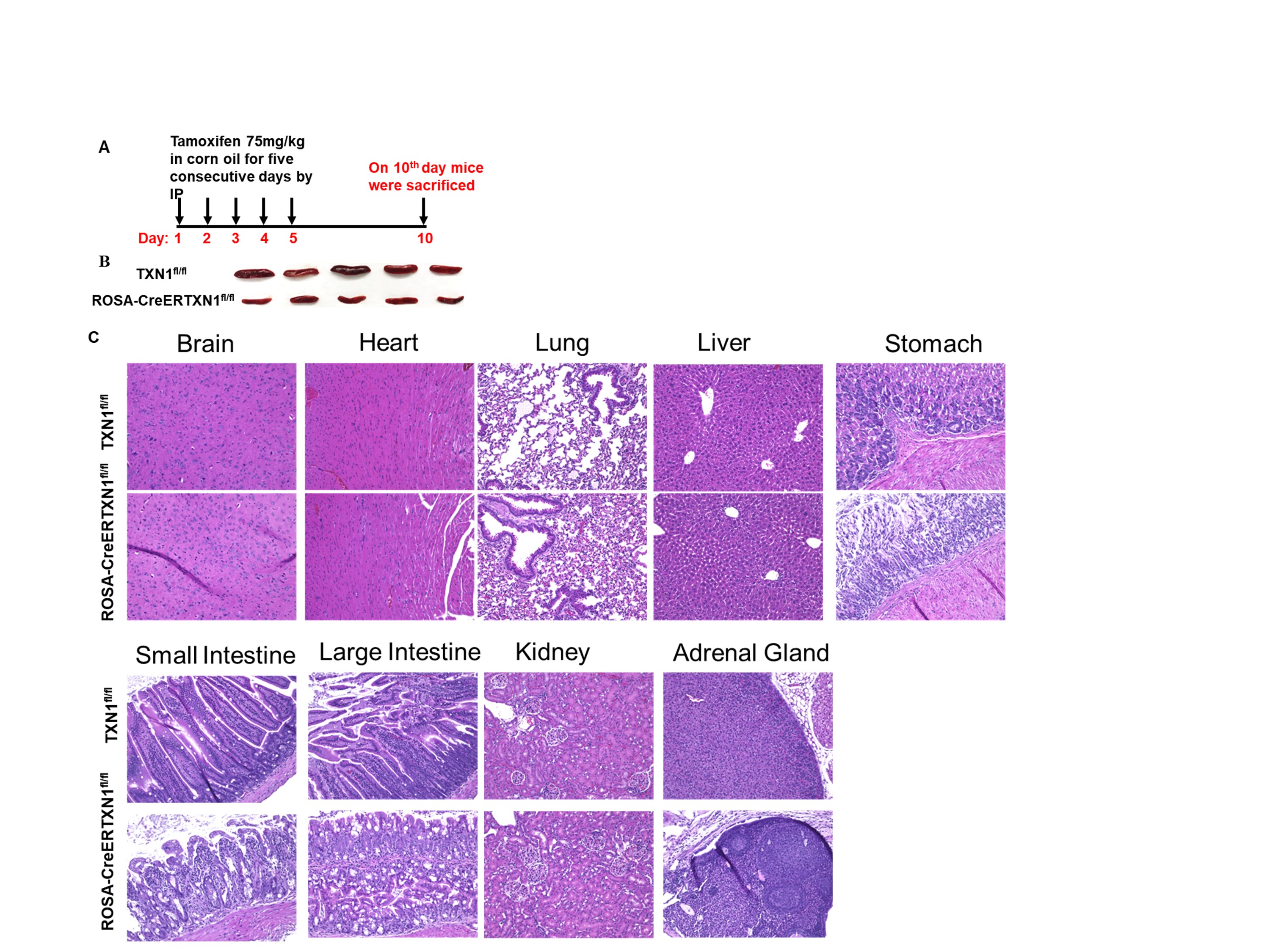

Supplement: Supplementary file 1 — Additional file 1: Figure S1. deletion of Thioredoxin leads to significant effects on different organs in mice. A) Schematic representation of tamoxifen injection in mice carrying the ROSA-Cre transgene to develop an inducible model for thioredoxin deletion. TXN1fl/fl (control) and ROSA-CreER-TXN1fl/fl mice were given TAM (75mg/kg) i.p. daily for 5 days. Mice were sacrificed at day 10. B) Representative photographs of spleens. TXN1fl/fl (control) and ROSA-Cre-TXN1fl/fl mice were treated with TAM (75mg/kg) i.p. daily for 5 days. Mice were sacrificed at day 10 and spleens were harvested. C) Histological sections (H&E staining) of the major organs of TXN1fl/fl (control) and ROSA-Cre-TXN1fl/fl mice, including Brain, heart, Lung, liver, stomach, small intestine, large intestine , kidney, and adrenal gland, No appreciable inflammatory response, cell degeneration, necrosis, or embolism was detected between TXN1fl/fl (control) and ROSA-Cre-TXN1fl/fl mice, Scale bar = 100 um. [file 40164_2022_329_MOESM1_ESM.jpg]

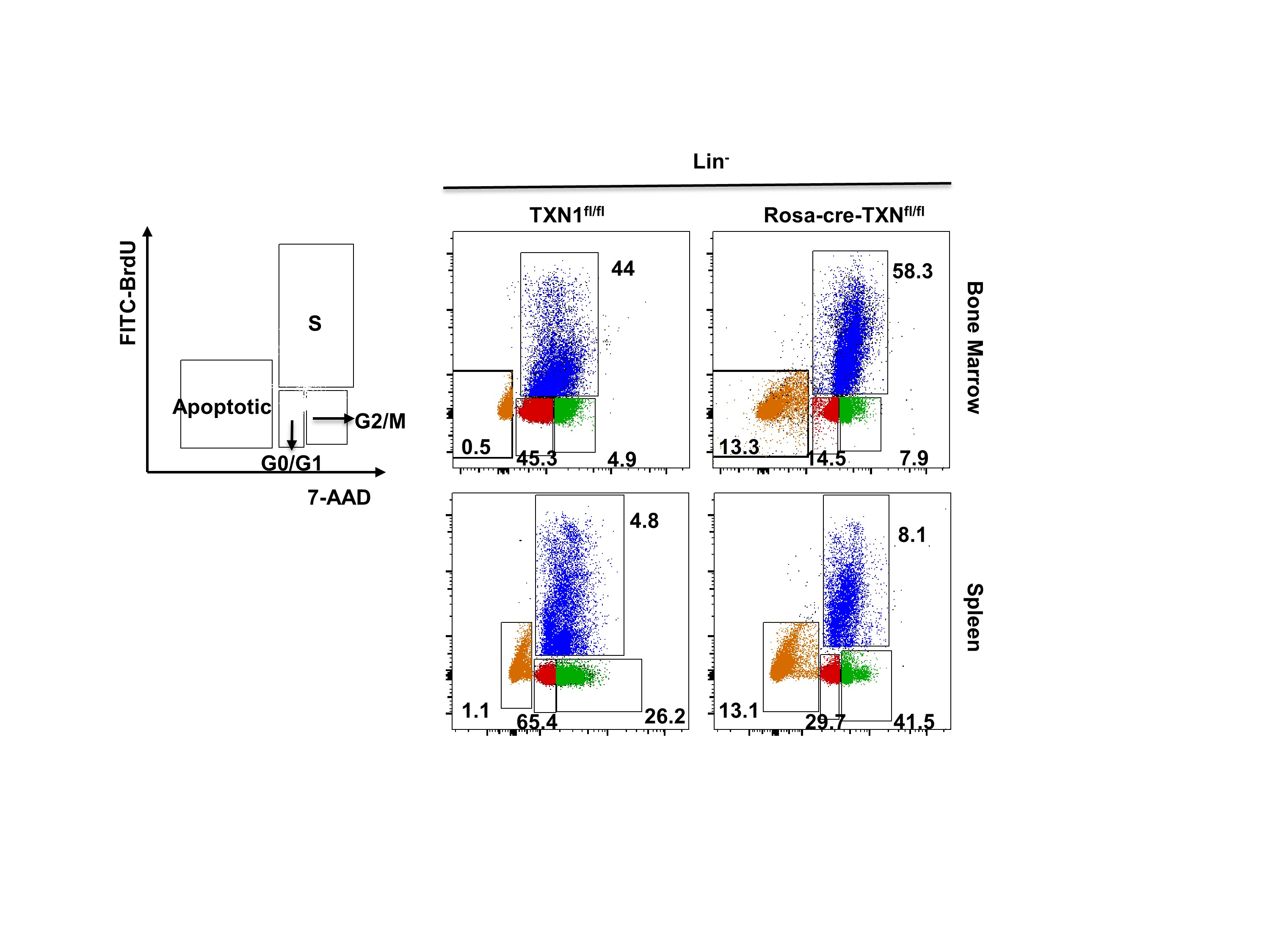

Supplement: Supplementary file 2 — Additional file 2: Figure S2. Conditional knockout of thioredoxin causes apoptosis and cell cycle activation of HSCs. LSK cells in mice were labeled with BrdU in vivo, followed by FACS analysis to assess the cell cycle profile, Brdu FITC and 7-AAD. Data in the two center panels were displayed as a histogram on the right. Data were representative of three independently performed experiments. [file 40164_2022_329_MOESM2_ESM.jpg]

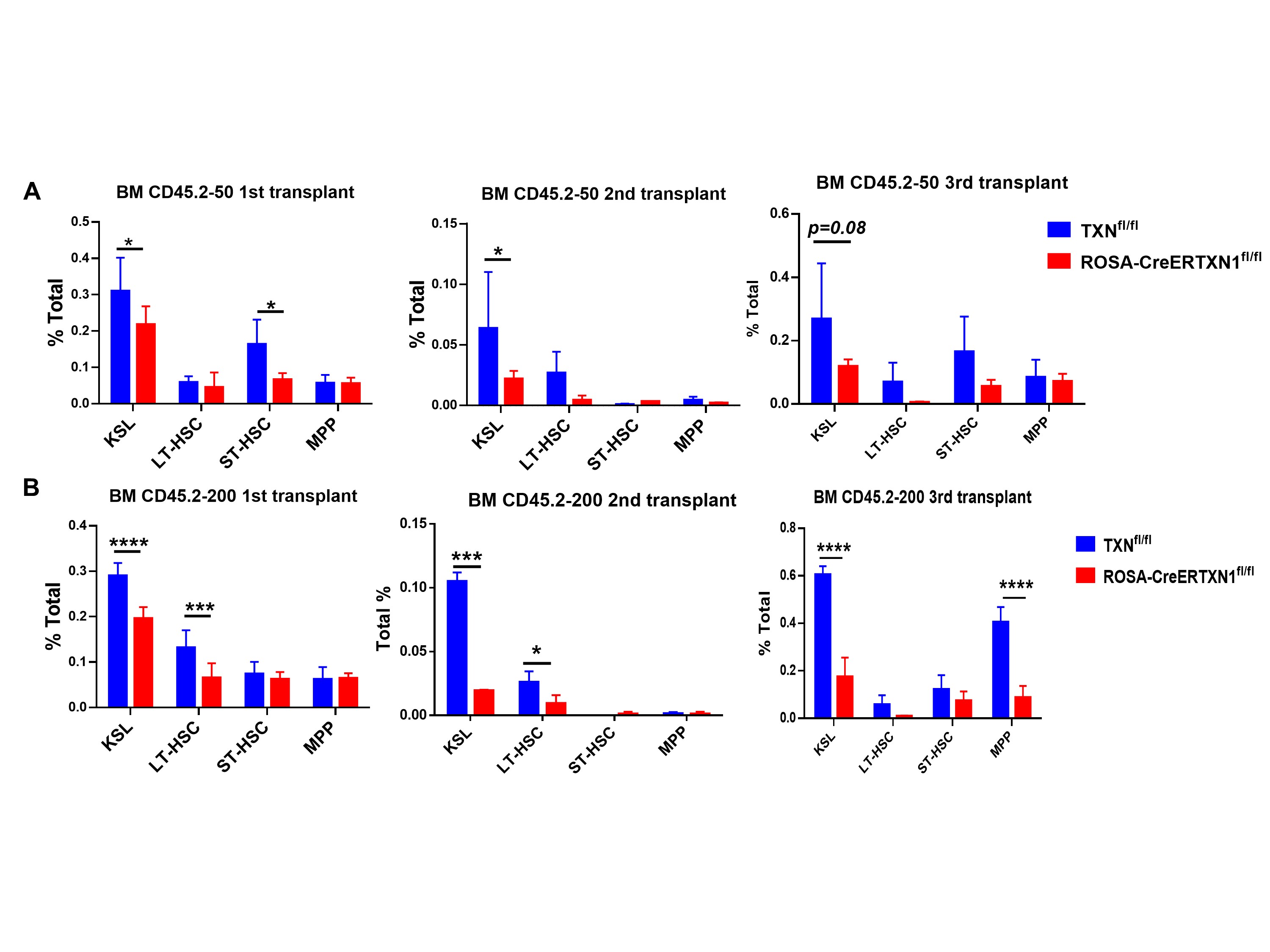

Supplement: Supplementary file 3 — Additional file 3: Figure S3. Conditional knockout of thioredoxin leads to significant suppression of hematopoiesis. A-B) The 2nd transplant recipient mice were sacrificed at 4 months after transplant. Bone marrow cells were harvested and injected into third, lethally irradiated (9.5Gy total body irradiation) CD45.1 C57bl/6J mice (5 x 10^6^ cells per mouse). Peripheral blood CD45.2 cells derived from TXN1fl/fl (control) and ROSA-CreER-TXN1fl/fl mice were measured at the indicated time-points in the tertiary transplant recipient mice (months) (5 x 10^6^cells per mouse). [file 40164_2022_329_MOESM3_ESM.jpg]

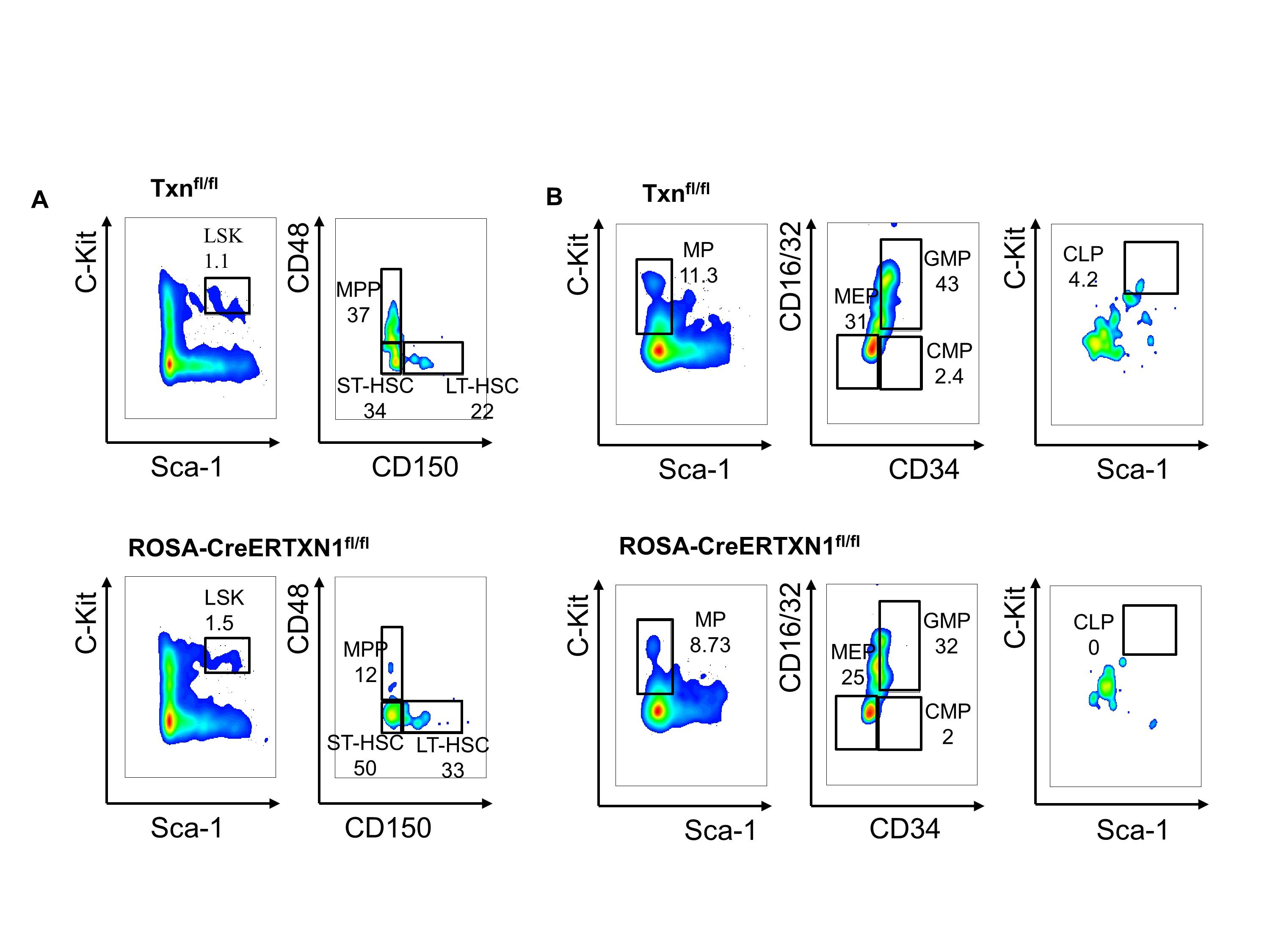

Supplement: Supplementary file 4 — Additional file 4: Figure 4. Gating strategy of bone marrow hemopoietic stem cells of TXN1fl/fl(control) and ROSA-CreER-TXN1fl/fl mice. The gating strategy was performed as follows: A) LSK cells: Lin- Scal+ C-Kit+; LT-HSC: Lin- Scal+ C-Kit+ CD150+ CD48-; ST-HSC: Lin- Scal+ C-Kit+ CD150- CD48-; MPP: Lin- Scal+ C-Kit+ CD150- CD48+. B) MP: Lin- Scal- C-Kit+; GMP: Lin- Scal- C-Kit+ CD16/32+ CD34+; CMP: Lin- Scal- C-Kit+ CD16/32- CD34+; MEP: Lin- Scal- C-Kit+ CD16/32- CD34-; CLP: Lin- Scal+ C-Kit+ CD127+. [file 40164_2022_329_MOESM4_ESM.jpg]

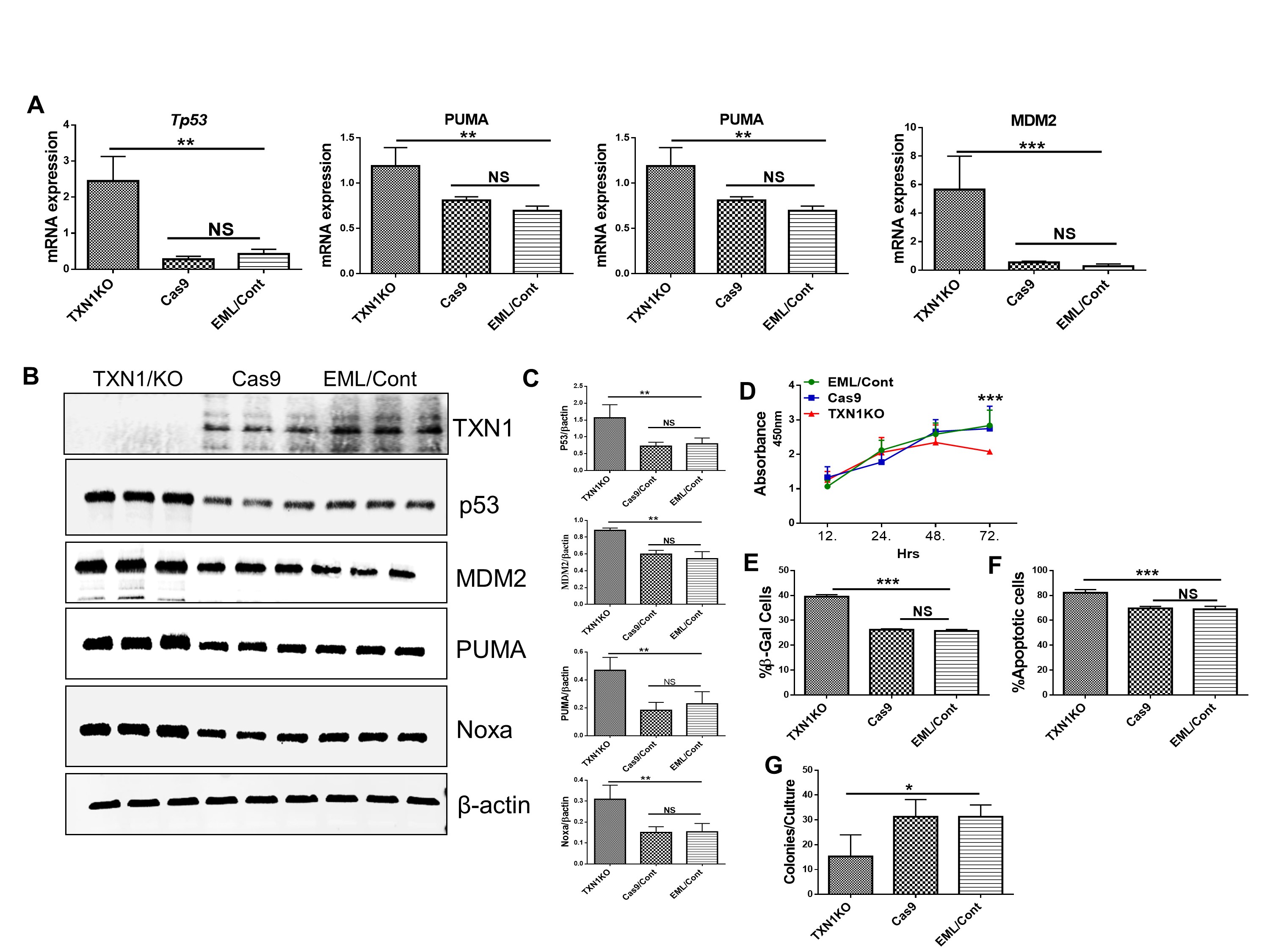

Supplement: Supplementary file 6 — Additional file 6: Figure S5. TXN1 deletion up-regulates P53 signaling pathways and impairs the proliferation and survival of EML cells. A: p53 and p53 downstream pathways: EML cells were transduced with TXN1 specific CRISPR/cas9 or Cas9 control vector. P53, MDM2, PUMA and Noxa mRNA expression in EML cell lines were quantified using qPCR (n=3 Mean+/-SEM or SD). B: Western blot analyses of P53 and P53 family expression in EML cell lines after deletion of TXN. protein quantity was compared to β-actin protein as housekeeping protein. C: quantification of P53, MDM2, PUMA and Noxa protein expression in relative to β-actin housekeeping protein. D) Knocking out TXN1 inhibits EML cells proliferation as detected by BrdU cell proliferation assay. EML control cells, EML Cas9 control cells and EML TXN1 KO cells were seeded at 5000 cell/well in 96well plate and incubated at different time points. Then, 10 μM BrdU was added to the cells and incubated for 4 hr. (E-F) respectively represent beta-gal expression and Annexin by flow cytometry staining in EML control cells, EML Cas9 control cells and EML TXN1 KO cells. G) In vitro cultured colony forming unit granulocyte macrophage (CFU-GM) in EML control cells, EML Cas9 control cells and EML TXN1 KO cells. Cells (EML/TXN1KO, EML/Cas9control and EML/control) were plated at 2X104/dish in Meth3434 semi-solid medium and incubated for 10-12 days. [file 40164_2022_329_MOESM6_ESM.jpg]

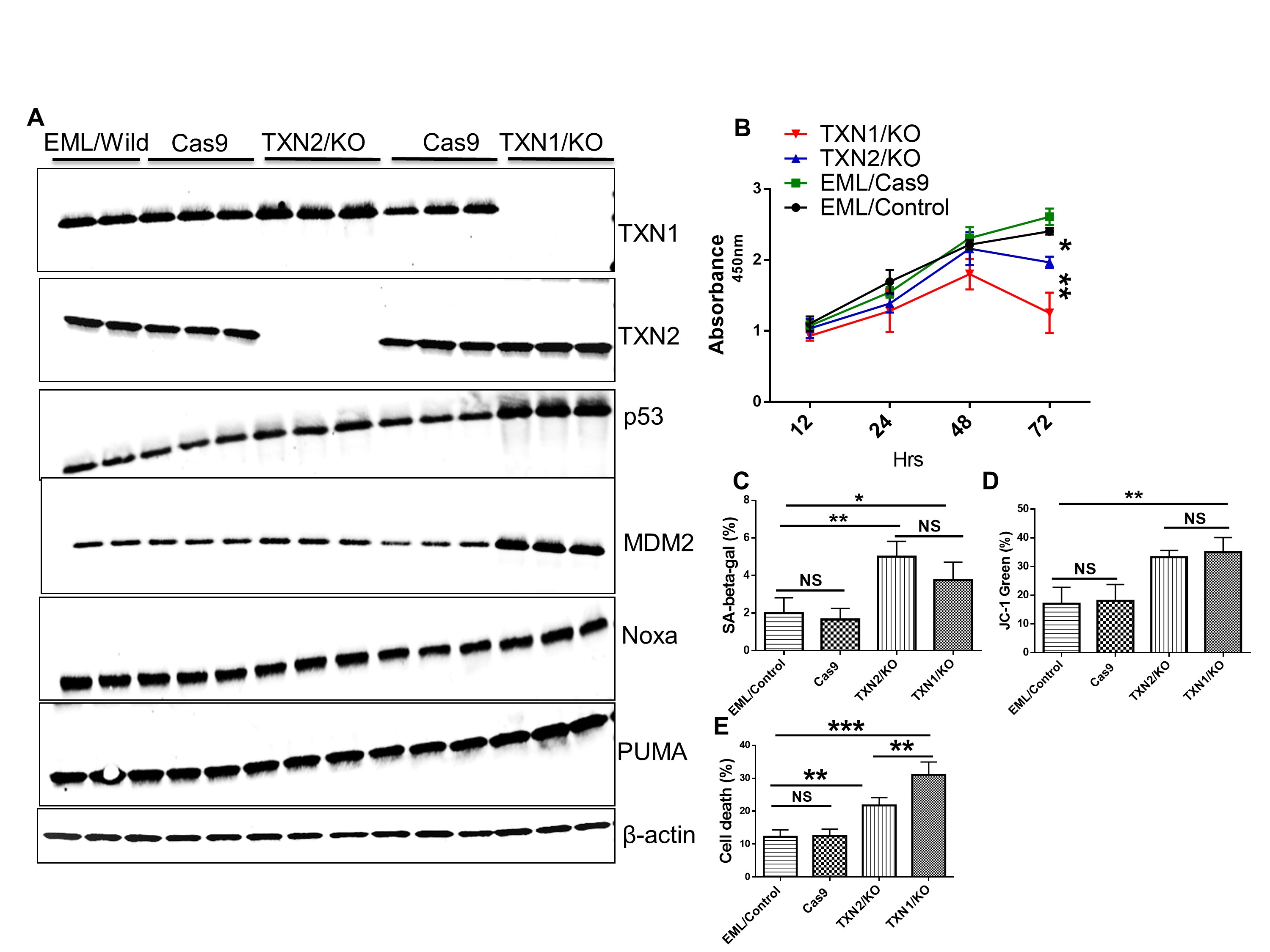

Supplement: Supplementary file 7 — Additional file 7: Figure S6. TXN2 deletion impairs the proliferation and survival of EML cells. A) EML cells were transduced with TXN1 or TXN2 specific CRISPR/cas9 or Cas9 control vector. p53 and p53 family expression in EML cell lines after deletion of TXN1 or TXN2 were quantified using immunoblot, protein quantity was compared to β-actin protein as housekeeping protein. (n=3 Mean+/-SEM or SD). B) Knocking out TXN1 inhibits EML cells proliferation as detected by BrdU cell proliferation assay. EML control cells, EML Cas9 control cells, EML TXN1 KO and EML TXN2 KO cells were seeded at 5000 cell/well in 96well plate and incubated at different time points. Then, 10 μM BrdU was added to the cells and incubated for 4 hr. C-E) respectively represent beta-gal expression, JC-1 and Annexin by flow cytometry staining in EML control cells, EML Cas9 control cells, EML TXN2 cells and EML TXN1 KO cells. [file 40164_2022_329_MOESM7_ESM.jpg]

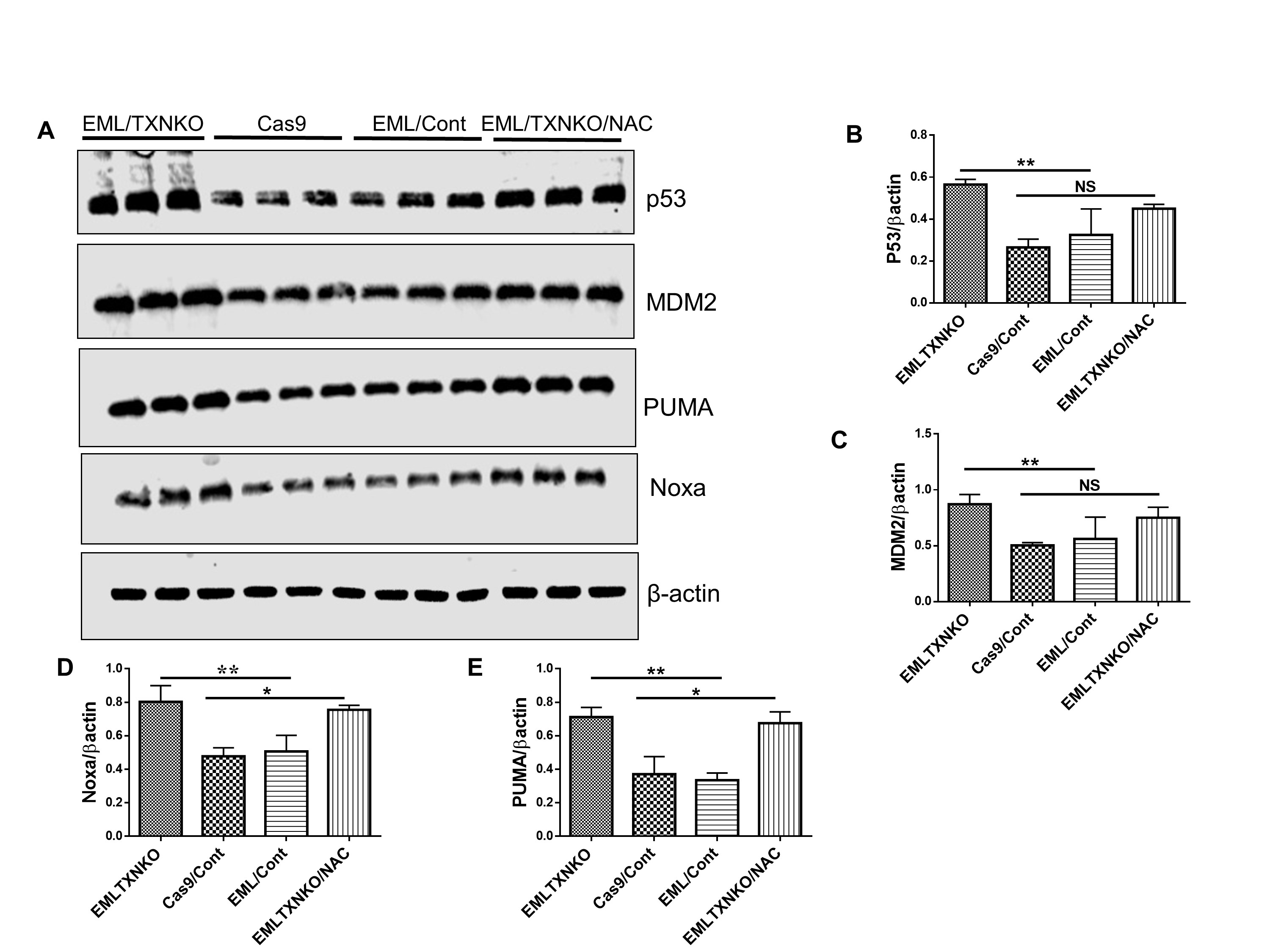

Supplement: Supplementary file 8 — Additional file 8: Figure S7. N-acetylcysteine supplement unable to normalize the expression level of P53 in EML cell lines. A) Representative western blots displaying P53 and P53 family member protein expressions in EML control, EML/TXN1KO and EML/TXN1KO treated with 250uM NAC. Beta-actin was used as a loading control. B-E) Western data was quantified using ImageJ software and normalized with beta-actin levels. Data represented mean±SD. [file 40164_2022_329_MOESM8_ESM.jpg]

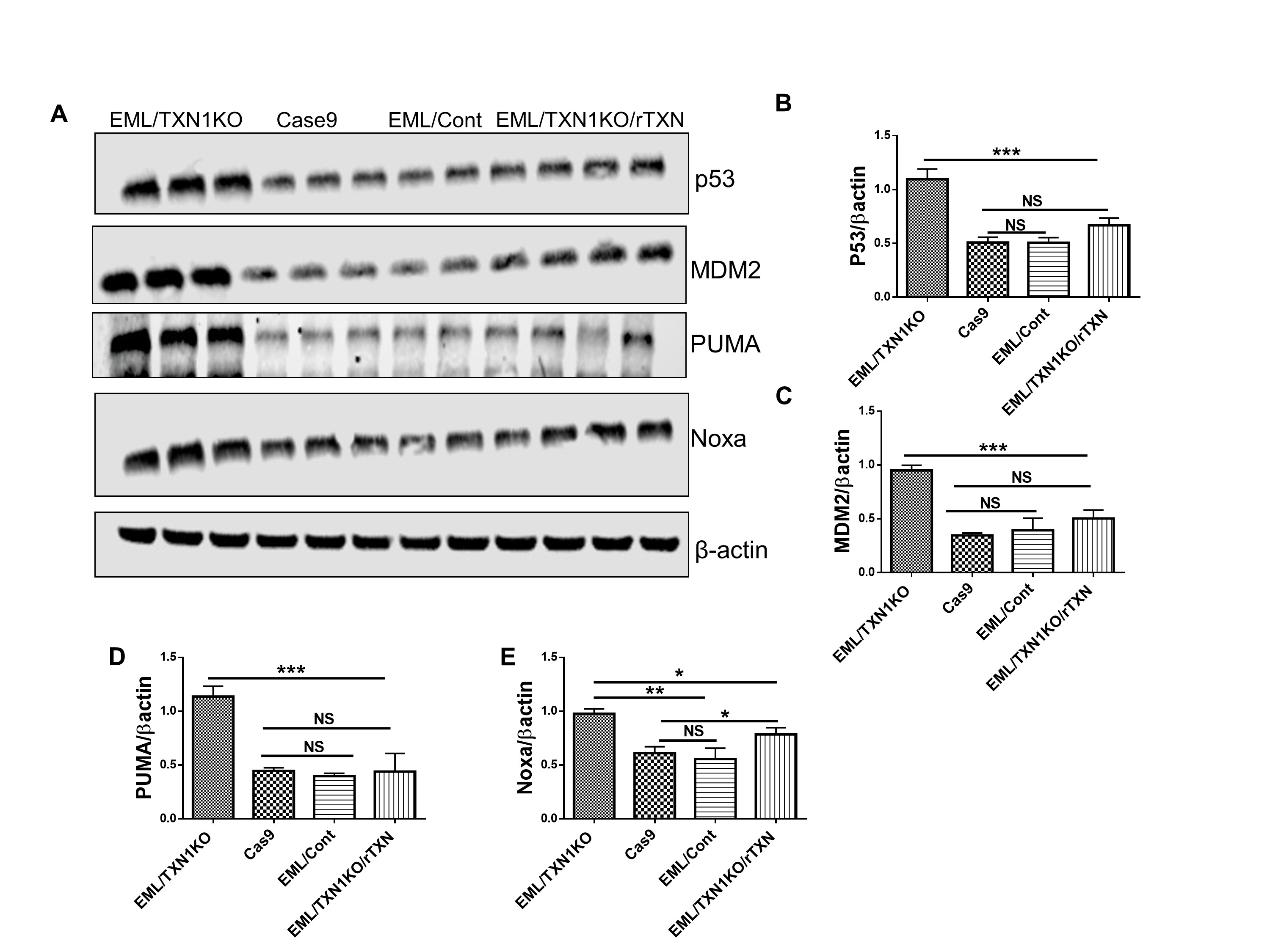

Supplement: Supplementary file 9 — Additional file 9: Figure S8. rTXN1 supplement downregulated P53 protein expression in EML/TXN1KO cells. A) Representative western blots displaying P53 and P53 family member protein expressions in EML control, EML/TXN1KO and EML/TXN1KO treated with rTXN1 32ug/ml. Beta-actin was used as a loading control. B-E) Western data was quantified using ImageJ software and normalized with beta-actin levels. Data represented mean±SD. [file 40164_2022_329_MOESM9_ESM.jpg]

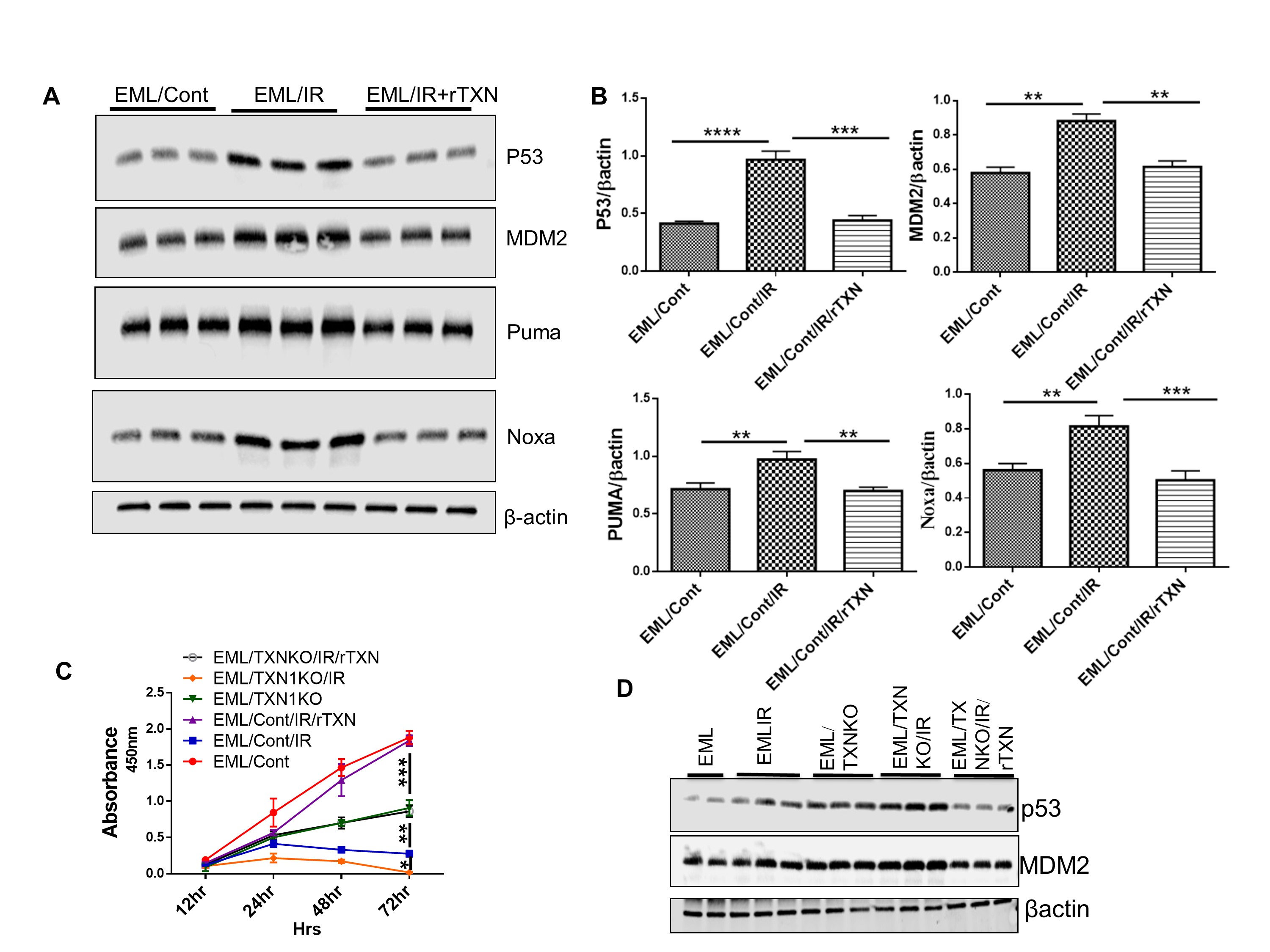

Supplement: Supplementary file 10 — Additional file 10: Figure S9. radiation enhanced apoptotic-signaling pathways in EML cells. A) Representative western blot analyses of P53 and P53 family genes expression in response to 5Gy radiation in EML cell lines and supplied with rTXN1 for 24hrs. B) Western data was quantified using ImageJ software and normalized with beta-actin levels. Data represented mean±SD. C) BrdU cell proliferation. EML control and EML TXN1 KO cells were irradiated with 5Gy, and plated in 96well plate/5000 cells/well and incubated with or without recombinant TXN1 for different time points as indicated in the figure. Plate was read at dual wavelength 450/550 nm. Data was plotted as mean ± SEM (n = 3). D) EML control and EML TXN1 KO cells were irradiated with 5Gy, and treated with or without recombinant TXN1. Cells lyses were collected after 24hrs of radiation and β-actin was used as internal housekeeping protein. [file 40164_2022_329_MOESM10_ESM.jpg]
